# Analysis of SARS-CoV-2 Population Genetics from Samples Associated with Huanan Market and Early Cases Identifies Substitutions Associated with Future Variants of Concern

**DOI:** 10.3390/v15081728

**Published:** 2023-08-12

**Authors:** Xiaofeng Dong, Julian A. Hiscox

**Affiliations:** 1 Institute of Infection, Veterinary and Ecological Sciences, Faculty of Health and Life Sciences, University of Liverpool, Liverpool L3 5RF, UK; xiaofeng@liverpool.ac.uk; 2 Health Protection Research Unit in Emerging and Zoonotic Infections, Liverpool L69 7BE, UK; 3 A*STAR Infectious Diseases Laboratories (A*STAR ID Labs), Agency for Science, Technology and Research (A*STAR), Singapore 138648, Singapore

**Keywords:** SARS-CoV-2, Huanan Market, Minor variation

## Abstract

SARS-CoV-2 began spreading through human-to-human transmission first within China and then worldwide, with increasing sequence diversity associated with time and the further spread of the virus. The spillover events in the Huanan market were associated with two lineages of SARS-CoV-2 (lineages A and B). Infecting virus populations and those in infected individuals consist of a dominant genomic sequence and minor genomic variants; these latter populations can indicate sites on the genome that may be subject to mutational changes—either neutral or advantageous sites and those that act as a reservoir for future dominant variants—when placed under selection pressure. The earliest deposited sequences with human infections associated with the Huanan market shared very close homology with each other and were all lineage B. However, there were minor genomic variants present in each sample that encompassed synonymous and non-synonymous changes. Fusion sequences characteristic of defective RNA were identified that could potentially link transmission chains between individuals. Although all the individuals appeared to have lineage B as the dominant sequence, nucleotides associated with lineage A could be found at very low frequencies. Several substitutions (but not deletions) associated with much later variants of concern (VoCs) were already present as minor genomic variants. This suggests that low-frequency substitutions at the start of a pandemic could be a reservoir of future dominant variants and/or provide information on potential sites within the genome associated with future plasticity.

## 1. Introduction

Early cases of the SARS-CoV-2 outbreak were associated with the Huanan Wholesale Seafood market (Huanan market) [1,2]. Genetic and epidemiological analyses have provided further evidence that the market was the epicenter of the COVID-19 pandemic, resulting in sustained chains of human-to-human transmissions [3,4]. These analyses have suggested that the spillover events at the market were likely over a constrained time period—with a minimum of two successful spillovers leading to the establishment of the transmission of the Pango-assigned Lineages B and A in the human population—as well as potentially leading to other spillovers that led to dead ends [3]. Lineage A is represented by the Wuhan/WH04/2020 sequence and shares two nucleotides (positions U8782 in ORF1ab and C28144 in ORF8) with certain bat coronaviruses (RaTG13 and RmYN02). However, for lineage B, different nucleotides are present at those sites—8782C and 28144U—represented by the Wuhan-Hu-1 strain.

As the pandemic progressed, and SARS-CoV-2 spread through human-to-human transmission both within China and then globally, the viral sequences became more and more diverse, with selection pressure acting on those with a fitness advantage including increased transmissibility and immune evasion. One of the first set of changes that had a genotype to phenotype difference and to gain dominance was the P323L substitution in NSP12 (the RNA-dependent RNA polymerase) and the D614G substitution in the spike protein [5,6].

Coronaviruses have a great propensity for recombination, and a natural consequence of this is the presence of insertions and deletions, recombinant genomes, and defective RNAs. Early in the COVID-19 pandemic, recurrent deletions were identified in the spike gene of SARS-CoV-2 during persistent infection in an immunocompromised host [1,7,8]. Subsequently, several Variants of Concern have been postulated to have arisen in persistently infected and/or immunocompromised hosts, and recombination between different variants has been identified in the synthesis of these VoC genomes [7].

The sequencing information of viral populations within an individual can provide two useful markers: The first is the dominant genome sequence—this is representative of the most abundant sequence in a sample. The second is the minor genomic variants present, which are viral sequences that have a lower abundance than the same site on the dominant genome sequence. These minor genomic variants may contain synonymous and/or non-synonymous (amino acid) substitutions that confer an advantage under selective pressure or affect the viral load and disease phenotype [9,10].

For patients associated with the Huanan market and early cases, the dominant SARS-CoV-2 genome sequences were found to be over 99.9% identical with each other [1,7,8]. In this study, publicly available sequence data were used to identify and investigate minor genomic variants at the start of the COVID-19 pandemic. Although the sequencing approaches were likely focused on recovering the dominant genome sequence, minor genomic variants could be identified. The data indicated that genetic variability was present, and amino acid substitutions associated with future VoCs and emerging lineages such as the P323L and D614G changes were identified. The data indicated that lineage B was dominant and low levels of lineage A identified it as a minor genomic variant.P

## 2. Materials and Methods

### 2.1. Consensus Genome and Minor Variations

The sequencing reads of samples S1–S16 were collected from four published works and the NCBI database (Table 1). All of these samples were sourced from Wuhan, China and the total RNA was extracted from bronchoalveolar lavage (BAL) fluid, followed by sequencing using an Illumina sequencing platform. Using this published raw sequencing information, the consensus genome sequences and minor genomic variants of these samples were generated as per our previous description [9,11]. Hisat2 v2.1.0 [12] was used to map the trimmed reads on the human reference genome assembly GRCh38 (release-91), downloaded from the Ensembl FTP site. The unmapped reads were extracted by bam2fastq (v1.1.0) and then mapped onto a known SARS-CoV-2 genome (GenBank sequence accession: NC_045512.2), using Bowtie2 v2.4.1 [12] by setting the options to parameters “—local -X 2000 —no-mixed”. Reads with a quality score below 11 were removed from the SAM file using SAMtools v1.9 [13] with the ‘view -q 10’ parameters. Mapped reads that were not the primary alignment, supplementary alignment, or those without a mapped mate were also removed from the SAM file using SAMtools with the ‘view -F 2316’ parameters. The SAM file was then converted into a BAM file using SAMtools with the ‘view -Sb’ parameters. This BAM file was further sorted using SAMtools with the ‘sort’ parameter. After that, the PCR and optical duplicate reads in the bam files were discarded using MarkDuplicates in the Picard toolkit v2.18.25 (http://broadinstitute.github.io/picard/, accessed on 1 May 2022) with the option of “REMOVE_DUPLICATES=true”. The resultant Bam file was processed by Quasirecomb v1.2 [14] to generate a phred-weighted table of nucleotide frequencies with the default setting. This phred-weighted table of nucleotide frequencies was parsed with a custom perl script to generate a consensus genome sequence [11]. The consensus genome sequence was then used as a template in the second round of mapping to generate a reference genome sequence for all downstream analyses. Reads (unmapped on human genome) were realigned to the reference SARS-CoV-2 consensus genome sequence using Bowtie2 with the parameter “—local -X 2000 —no-mixed”. The Bowtie2 outputs were processed in the same way as above to generate a Bam file without read duplications. This Bam file was then processed using the diversiutils script in DiversiTools (http://josephhughes.github.io/btctools/, accessed on 1 May 2022) with the “-orfs” function to generate the number of amino acid change caused by the nucleotide deviation at each site in the protein. In order to distinguish the low-frequency variants from Illumina sequence errors, the diversiutils script used calling algorithms based on the Illumina quality scores to calculate a *p*-value for each variant at each nucleotide site [15]. The amino acid change was then filtered based on the *p*-value (<0.05) by removing the low-frequency variants from the Illumina sequence errors.

### 2.2. Insertion, Deletion, and Fusion

A Bam file was generated by realigning the reads (unmapped on the human genome) to the reference SARS-CoV-2 consensus genome sequence using Bowtie2 with the parameter “—local -X 2000 —no-mixed”. Reads with a quality score below 11 were removed from the SAM file using SAMtools v1.9 [13] with the ‘view -q 10’ parameters. Mapped reads that were not the primary alignment, supplementary alignment, or those without a mapped mate were also removed from the SAM file using SAMtools with the ‘view -F 2316’ parameters. The SAM file was then converted into a BAM file using SAMtools with the ‘view -Sb’ parameters. This BAM file was further sorted using SAMtools with the ‘sort’ parameter. After this, the PCR and optical duplicate reads in the bam files were discarded using MarkDuplicates in the Picard toolkit v2.18.25 (http://broadinstitute.github.io/picard/, accessed on 1 May 2022), with the option “REMOVE_DUPLICATES = true”. Insertion and deletion were then called by analysis of these resultant Bam files using FreeBayes (v1.3.5) [16] with the parameter of “—ploidy 1”. Variant filtering was carried out using the VCFfilter tool v1.0.1 [17] in vcflib, using a quality cutoff of 10.

Sequencing reads (unmapped on human genome) were aligned to the reference SARS-CoV-2 genome (GenBank sequence accession: NC_045512.2) with BWA-MEM (v0.7.17) [18]. Alignments from BWA-MEM were stored in SAM files. Samblaster v0.1.26 [19] was used to remove the duplicates in the SAM files with the ‘—excludeDups —addMateTags’ parameters. Subsequently, the SAM files were converted to the BAM format and sorted using SAMtools with the ‘sort’ parameter. Following this, the sorted BAM files were analysed by delly v1.0.3 [20] to identify fusion sites on the reference SARS-CoV-2 genome using the “call” function with the default settings. Only the fusion events that passed all the delly filters were reported.

## 3. Results and Discussion

To study the minor genomic variants of SARS-CoV-2 at the start of the COVID-19 pandemic, sequencing data from the first cases were analyzed to define the populations of minor genomic variants. This encompassed samples from patients with a reported association with the Huanan market or the earliest samples (samples collected in 2019) reported according to symptom onset date or deposition of the sequence. Data was identified from 16 patients (Table 1) from whom SARS-CoV-2 had been sequenced from bronchoalveolar lavage fluid using a meta-transcriptomic approach—in this case, on Illumina platforms. Undoubtedly the public health purpose of this sequencing was to identify the dominant genome sequence, rather than identifying viral sequence diversity, within a patient. Some of these sequenced samples had a relatively low sequencing depth, and this may have influenced base calling in assigning minor genomic variants at nucleotide positions on the virus genome. In order to improve the confidence in base calling for some cases with low read depths, a pipeline was applied that had been used to identify minor genomic variants in Ebola virus from clinical samples from patients with Ebola virus disease [9]. This pipeline excluded reads from contamination and PCR duplicates, and removed reads with low quality. The pipeline also distinguished low-frequency variants from Illumina sequencing errors, although Illumina platforms produce reads with very low error rates [21]. The pipeline used a base calling algorithm based on the Illumina quality scores to calculate a *p*-value for each variant at each nucleotide site [15], to best ensure the correct calling of a minor genomic variant at a low sequencing depth. For this study, patients were ordered by symptom-onset date and given a sample ID from S1 to S16 for ease of labeling. How this labeling relates to accession IDs, data deposition, and WHO IDs is described in Table 1.

**Figure 1:**
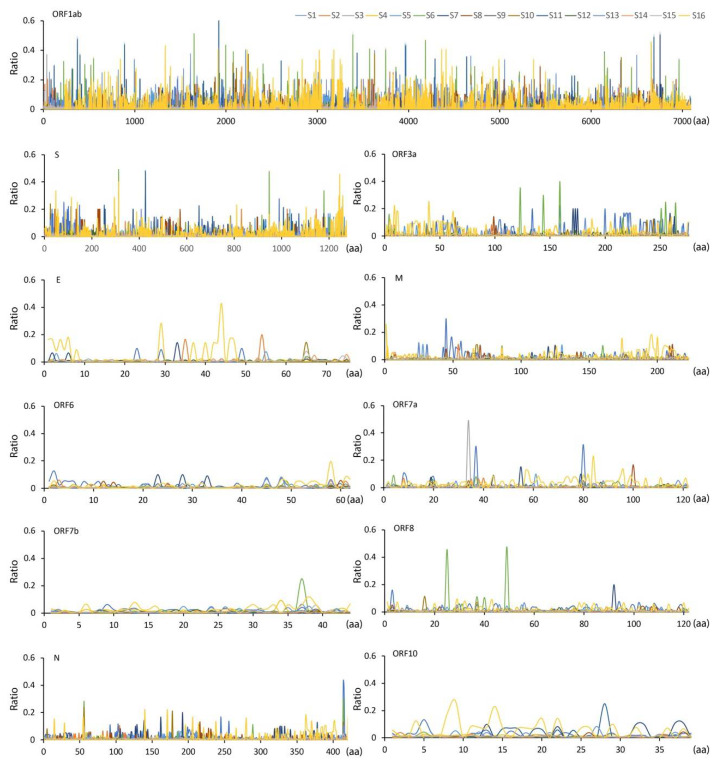
Map of minor genomic variants across the SARS-CoV-2 genome, showing non-synonymous substitutions for each gene from samples sequenced from 16 patients selected for this study. The position of each amino acid (aa) is shown on the X-axis and the Y-axis shows the ratio of that substitution. Note that this cannot be more than 49%, otherwise this would be the dominant genome sequence. The amino acid sites with coverage >=5 were shown. Each SARS-CoV-2 gene is indicated by its abbreviation where appropriate and in gene order along the genome from 5′ to 3′: ORF1ab, spike (S), ORF3A, envelope (E), membrane (M), ORF6, ORF7a, ORF7b, ORF8, nucleoprotein (N), and ORF10.

Minor genomic variants were identified across all genes in SARS-CoV-2 for each patient (Figure 1 for combined patients showing non-synonymous changes and Figure S1 for individual patients showing non-synonymous changes). The data indicated that for minor genomic variants in some genes, amino acid substitutions at specific sites were tolerated, whereas in other genes these were less frequent—including the envelope (E), membrane (M), ORF6, ORF7b, and ORF10. At the level of individual patients there were some patients with very low population diversity in terms of SARS-CoV-2 minor genomic variants, including patients S9, S12, and S14 (Figure S1). There were higher-frequency substitutions in SARS-CoV-2 from some patients; for example, in SARS-CoV-2 from patient S6, there were two substitutions (C25R and V49I) in ORF8 that were approximately 40%. 

**Figure 2:**
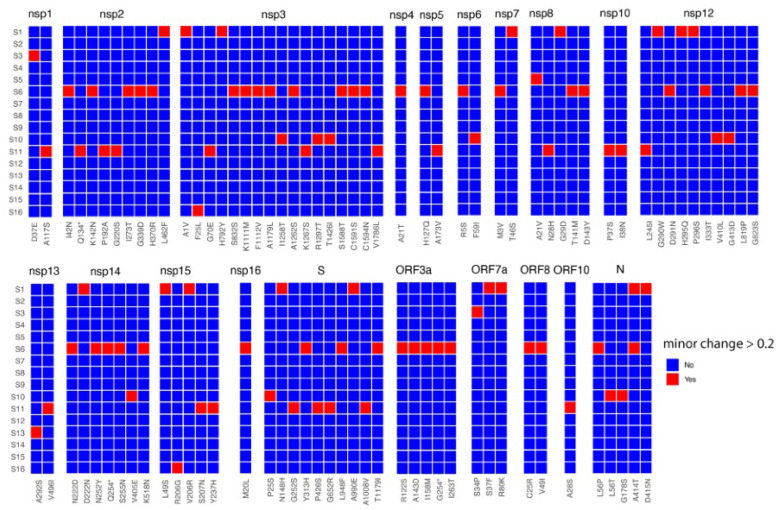
Heat map of non-synonymous changes at the minor genomic variant level in SARS-CoV-2 that have a threshold of between 20 and 49% in the 16 patients (Y-axis). The panel is divided into each of the SARS-CoV-2 proteins and substitutions are shown on the X-axis. Amino acid sites with coverage ≥10 are shown.

To investigate abundant minor genomic variants and the implications of amino acid substitutions and phenotype, a frequency cut off between the 20 and 49% threshold was considered. With this threshold, there were four patients that had a greater number of minor variant genomes in SARS-CoV-2: patients S1, S6, S10, and S11 (Figure 2). Several of the SARS-CoV-2 minor genomic variants sampled from these patients had premature stop codons. For example, premature stop codons were identified in SARS-CoV-2 from patient S11 in NSP2 and in NSP14 and ORF3A from patient S6. As these were present as minor genomic variants, the activity of the wildtype protein may have been impacted by a pool of aberrantly functioning proteins—as was described for the Ebola virus in patients with Ebola virus disease [9].

**Figure 3:**
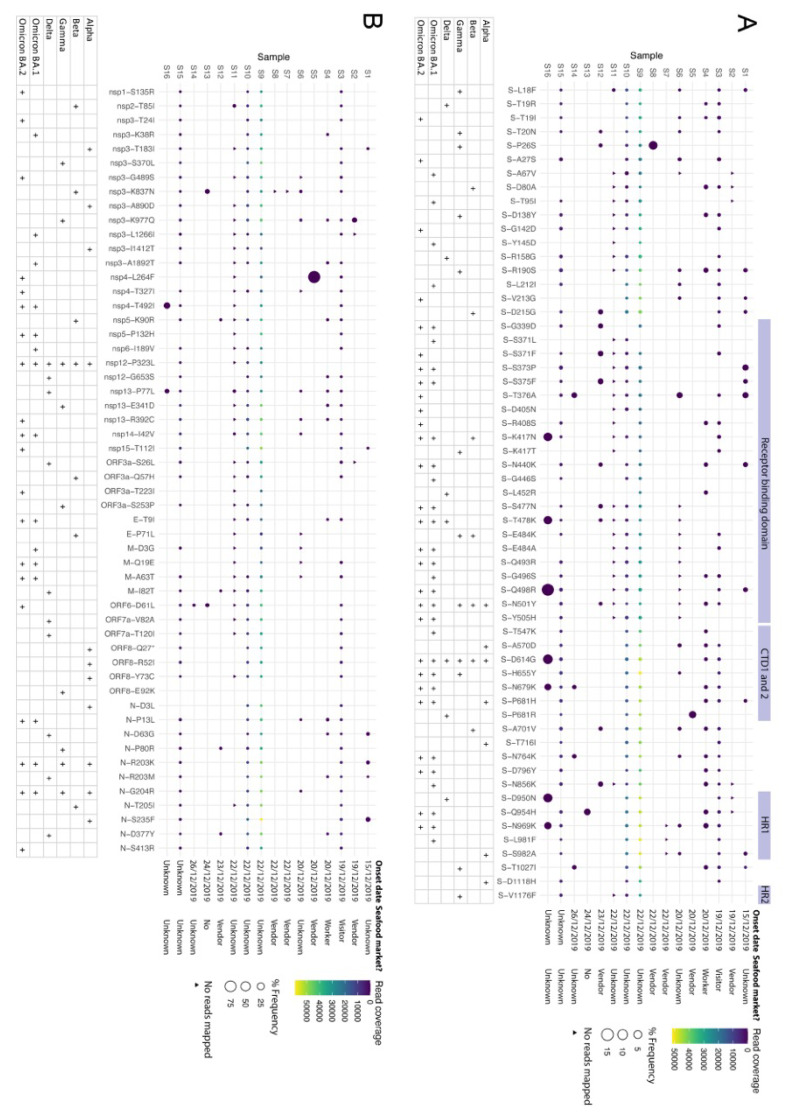
Non-synonymous substitutions in the minor genomic variants of SARS-CoV-2 from each of the 16 selected patients, focusing on sites that define VoCs (https://covariants.org/variants, accessed on 1 May 2022) in the spike protein (**A**) and other regions of the genome (**B**). Indicated for each protein is the substitution (X-axis) and its position, as well the read coverage and frequency within the viral population for each patient. Features of interest in the spike protein are indicated. Shown also is which VoC the substitution is associated with. Illumina sequence errors were removed as described in the methods.

Interestingly, several minor genomic variants were identified in the spike protein (Figure 3A, using *p*-value) and other viral proteins (Figure 3B, using *p*-value) that were subsequently found in different VoCs. These were not uniform in position or frequency in SARS-CoV-2 sampled from different patients. Nevertheless, the data indicated that in terms of amino acid substitutions, the hallmarks of the WHO VoCs (https://covariants.org/variants, accessed on 1 May 2022) were already present at the start of the pandemic or indicated a tolerability of substitutions at these positions in SARS-CoV-2 from some patients. This included the P323L substitution in NSP12 in SARS-CoV-2 minor genomic variants from patients S3, S9, S10, and S15 (Figure 3B); although below 5%, at the level of a minor genomic variant in these patients, data suggests that this substitution is under strong selection pressure and can become dominant in an infection within days [22]. Minor genomic variants of SARS-CoV-2 from patient 16 had several substitutions of between a frequency of 5 and 15%. These included substitutions in the spike protein K417N, associated with the ‘Delta plus’ variant; T478K, associated with Delta; Q498R, associated with Omicron; D614G, first associated with an increase in transmission from the Wuhan reference sequence/virus [5]; and N679K, associated with Gamma and Omicron, which adds to the polybasic nature of the furin cleavage site. Most of these the substitutions were still detected even after applying a site coverage cutoff of at least ten or 100 (Figures S2 and S3).

The split in lineage A and lineage B variants for SARS-CoV-2 has been proposed to have been more likely in an intermediate animal host, with lineage B likely derived from lineage A [3]. Two scenarios may have been possible: First, lineage B was derived from lineage A in a single host and established a unique infection in another host, despite the likelihood of this being a mixed infection—with lineage A as the dominant sequence. Second, lineage B was derived from lineage A at the start of an infection in a host, became dominant, and spread from that animal with no mixing with animals infected with lineage A. In this study, the dominant genomes in SARS-CoV-2 from patients associated with Huanan market and the earliest SARS-CoV-2 cases were all assigned to lineage B. The sequence associated with lineage A at positions 8782 and 28,144 could be observed at a very low frequency as minor genomic variants in samples from patients with higher coverage at these two sites (Table 2).

The coexistence of two variants has been documented in previous research using a large population sample from the UK and worldwide [22]. The same individual can be infected by both the D614 and 614G variants in the spike protein, as well as the P323 and 323L variants in NSP12 with varying ratios—some of which have been observed to be 50:50. Infections containing two the variants P323 and 323L have also been observed in two non-human primate models: cynomolgus and rhesus macaques [22]. This does not necessarily imply that the individuals were infected with two different variants from separate sources—these could also be part of the same infecting virus population. Mutation events occurring within the host could also provide a source of new genomic variants. If these new mutations enable the virus to adapt to the host, they may be transmitted to other individuals as part of the infecting viral swarm. A model for the transmission of variant genomes indicates that the distribution of minor genomic variants and the dominant viral genome sequence for SARS-CoV-2 is dependent on selection pressure and the time post-infection at which a virus population is transmitted onwards to another individual [22]. The difference in non-synonymous and synonymous mutation frequencies between inoculation and the days post-infection in the two non-human primate models [22] are shown in Figures S4 and S5. Most of the non-synonymous and synonymous mutations are the same between inoculation and the days post-infection. Some of the non-synonymous frequencies increased with time compared to inoculation, while the differences for synonymous mutation frequencies were few. This suggests that synonymous mutations have less of an effect while in the host.

**Figure 4:**
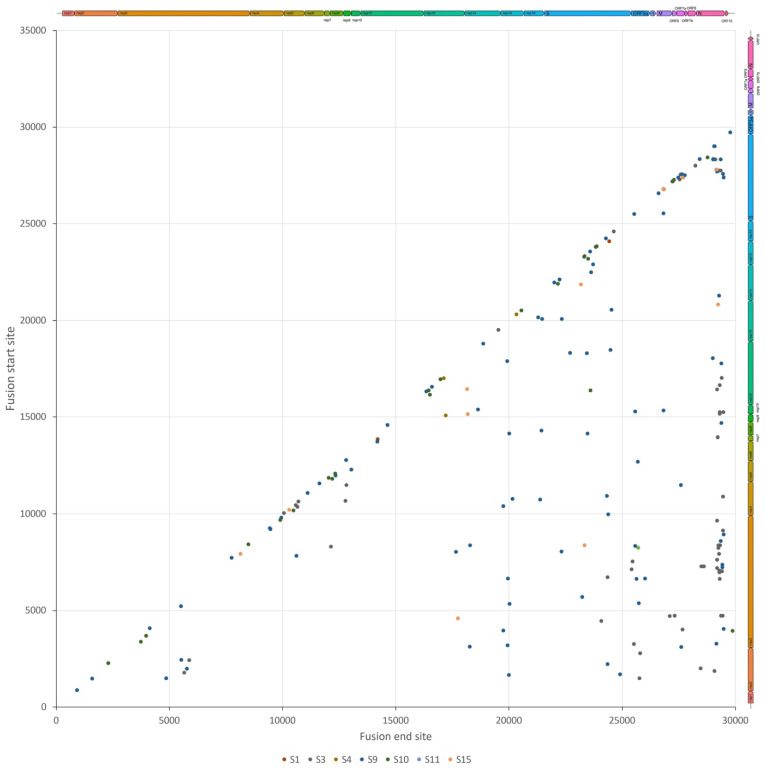
Virus genome position of the start of the fusion site (Y-axis) plotted against the end fusion site (X-axis) to show the fusion events along the SARS-CoV-2 genome. Recombination events that generate subgenomic mRNAs as a result of discontinuous transcription during negative strand synthesis were excluded.

Minor genomic variants may provide the possibility of establishing potential transmission chains between different patients. For example, the minor genomic variants of SARS-CoV-2 in patients S3, S9, and S10 appeared to have a similar profile of non-synonymous substitutions, suggesting a relationship between them (although we note these were low frequency, revealed by a high read depth). To investigate these further, common fusion sites were identified in the population of SARS-CoV-2 in each patient (Figure 4). The formation of fusion sites between disparate parts of the genome is common in coronaviruses due to their high rates of recombination inside coronavirus-infected cells [23], as well as the mechanism of subgenomic mRNA synthesis [24]—this process results in the formation of defective RNAs. Subgenomic mRNAs were computationally identified and removed from this analysis of the sequencing data [25]. The deletion analysis pulled out two interesting aspects: The first is that patients S9, S10, and S3 had fusion sites in their SARS-CoV-2 genomes in common. Between S9 and S10, there were three sites in common, with fusions between nucleotides 2273 to 2307, 12076 to 12329, and 23795 to 23828. Between patients S3 and S9, there was one site in common in their SARS-CoV-2 genomes between nucleotides 2554 to 23583; this would suggest a potential common source between them. The second was that the fusion sites were most common within the nucleoprotein gene, and between this and other genes. Loss of nucleoprotein is unlikely to be tolerated due to the functions involved in virus replication [26]. Deletions associated with VoCs were not found in these samples, suggesting that these developed much later than the potential amino acid substitutions (Table 3).

This study identified potentially interesting sequence features in the SARS-CoV-2 population at the start of the COVID-19 pandemic. Open-source data from samples sequenced by different groups indicated the presence of minor genomic variants; these contained substitutions that were under selection pressure much later in the outbreak. Certain amino acid substitutions within these minor genomic variants were associated with future VoCs—such as those associated with alpha, delta, and omicron variants of concern. Predicting the emergence of VoCs is a crucial goal in determining their implications for therapeutic treatment regimens and vaccination. Such approaches have in part relied on in vitro evolution studies [27] and rapid experimental work once a VoC has emerged. Our analysis does not necessarily suggest that these minor genomic variants with these VoC-associated substitutions were spread as the outbreak progressed; however, this work does indicates that capturing early sequence information and understanding the genetic diversity in viral populations may provide an insight into what sites may potentially mutate as an outbreak progresses. The COVID-19 pandemic and concomitant sequencing of SARS-CoV-2 has provided us with the first extensive guide to the origin and evolution of a virus in real time. This work suggests that analysis of minor genomic variants and the identification of variable sites at the start of a viral outbreak can provide a partial playbook for the evolution of the virus. Such information can be used to inform the broad utility of medical countermeasures and challenges to immunity in the face of a virus that has the potential for great diversity. 

## Figures and Tables

**Table 1:** Information of 16 sequenced samples collected from NCBI.

Sample ID	Accession ID	SRA ID	WHO ID	ID in Article	Virus Strain	Lineage	Gender	Age	Onset Date	Collection Date	Wuhan Seafood Market	ICU	Sample	Sequencing Method	Publication Link
S1	SRX7705833	SRR11059945	-	-	nCov-RNA-3	B	male	40	15 December 2019	30 December 2019	-	yes	BAL	Illumina HiSeq 2500 paired end sequencing	https://doi.org/10.1093/cid/ciaa207 accessed on 1 May 2022
S2	SRX7730880	SRR11092063	WHO_S04	ICU-04	WIV02	B	male	32	19 December 2019	30 December 2019	Vendor	yes	BAL	Illumina HiSeq 3000 paired end sequencing	https://www.nature.com/articles/s41586-020-2012-7 accessed on 1 May 2022
S3	SRX7705834	SRR11059944	-	-	nCov-RNA-4	B	male	61	19 December 2019	1 January 2020	Visitor	yes	BAL	Illumina HiSeq 2500 paired end sequencing	https://doi.org/10.1093/cid/ciaa203 accessed on 1 May 2022
S4	SRX7636886	SRR10971381	WHO_S06	-	Hu-1	B	male	41	20 December 2019	26 December 2019	Worker	-	BAL	Illumina MiniSeq paired end sequencing	https://www.nature.com/articles/s41586-020-2008-3 accessed on 1 May 2022
S5	SRX7730884	SRR11092059	WHO_S08	ICU-10	WIV07	B	male	56	20 December 2019	30 December 2019	Vendor	yes	BAL	Illumina HiSeq 3000 paired end sequencing	https://www.nature.com/articles/s41586-020-2012-7 accessed on 1 May 2022
S6	SRX7705836	SRR11059942	-	-	nCov-RNA-6	B	male	56	20 December 2019	30 December 2019	-	yes	BAL	Illumina HiSeq 2500 paired end sequencing	https://doi.org/10.1093/cid/ciaa209 accessed on 1 May 2022
S7	SRX7730882	SRR11092061	WHO_S11	ICU-08	WIV05	B	female	52	22 December 2019	30 December 2019	Vendor	yes	BAL	Illumina HiSeq 3000 paired end sequencing	https://www.nature.com/articles/s41586-020-2012-7 accessed on 1 May 2022
S8	SRX7730883	SRR11092060	WHO_S12	ICU-09	WIV06	B	male	40	22 December 2019	30 December 2019	Vendor	yes	BAL	Illumina HiSeq 3000 paired end sequencing	https://www.nature.com/articles/s41586-020-2012-7 accessed on 1 May 2022
S9	SRX7705831	SRR11059947	-	-	nCov-RNA-1	B	female	49	22 December 2019	30 December 2019	-	no	BAL	Illumina HiSeq 2500 paired end sequencing	https://doi.org/10.1093/cid/ciaa205 accessed on 1 May 2022
S10	SRX7705832	SRR11059946	-	-	nCov-RNA-2	B	female	52	22 December 2019	30 December 2019	-	yes	BAL	Illumina HiSeq 2500 paired end sequencing	https://doi.org/10.1093/cid/ciaa206 accessed on 1 May 2022
S11	SRX7705835	SRR11059943	-	-	nCov-RNA-5	B	male	40	22 December 2019	30 December 2019	-	no	BAL	Illumina HiSeq 2500 paired end sequencing	https://doi.org/10.1093/cid/ciaa208 accessed on 1 May 2022
S12	SRX7730881	SRR11092062	WHO_S10	ICU-06	WIV04	B	female	49	23 December 2019	30 December 2019	Vendor	yes	BAL	Illumina HiSeq 1000 paired end sequencing	https://www.nature.com/articles/s41586-020-2012-7 accessed on 1 May 2022
S13	SRX7705837	SRR11059941	-	-	nCov-RNA-7	B	female	53	24 December 2019	1 January 2020	No	no	BAL	Illumina HiSeq 2500 paired end sequencing	https://doi.org/10.1093/cid/ciaa204 accessed on 1 May 2022
S14	SRX7705838	SRR11059940	-	-	nCov-RNA-8	B	male	41	26 December 2019	30 December 2019	-	no	BAL	Illumina HiSeq 2500 paired end sequencing	https://doi.org/10.1093/cid/ciaa210 accessed on 1 May 2022
S15	SRX8032203	SRR11454614	-	-	HBCDC-HB-01/2019	B	female	49	-	30 December 2019	-	-	BAL	Illumina MiSeq paired end sequencing	
S16	SRX8032205	SRR11454612	-	-	HBCDC-HB-04/2019	B	male	-	-	30 December 2019	-	-	sputum	Illumina MiSeq paired end sequencing	

BAL: bronchoalveolar lavage fluid.

**Table 2:** Substitutions of C8782U and U28144C that separated Lineage A and B. “NA” indicated that no sequencing read was mapped on the nucleotide site.

Sample	Position	Site Coverage	C8782U	U28144C
S01	8782	16	0.00%	0.00%
S02	8782	1	0.00%	0.00%
S03	8782	846	0.12%	0.07%
S04	8782	209	0.00%	0.00%
S05	8782	16	0.00%	0.00%
S06	8782	5	0.00%	0.00%
S07	8782	3	0.00%	0.00%
S08	8782	5	0.00%	0.00%
S09	8782	28619	0.16%	0.18%
S10	8782	1413	0.07%	0.19%
S11	8782	NA	NA	NA
S12	8782	82	0.00%	0.00%
S13	8782	9	0.00%	0.00%
S14	8782	63	0.00%	0.00%
S15	8782	2781	0.07%	0.27%
S16	8782	3	0.00%	0.00%

**Table 3:** Nucleotide insertions and deletions identified by freebayes.

Sample	Position	Gene	Reference	Alternative	Inserted Nucleotide	Deleted Nucleotide	Quality Score
S1	8084	nsp3	GAAAAACT	GAAAACT	-	A	4290.82
S1	18976	nsp14	CAACACA	CAAACACA	A	-	452.059
S6	8837	nsp4	ATA	AA	-	T	22.9267
S6	13884	nsp12	ATA	AA	-	T	43.2573
S6	13893	nsp12	TTG	TATG	-	A	21.4099
S11	2550	nsp2	TAAACCAACCAT	TACCAACCAT	-	AA	1276.76
S11	6023	nsp3	TATCCAA	TATCAA	-	C	1001.59
S11	10024	nsp4	ACA	ATCA	T	-	330.538

## Data Availability

All data analysed in this study were retrieved published works and the NCBI database as described in the Materials and Methods section.

## Supplementary Materials

The following supporting information can be downloaded at: https://www.mdpi.com/article/10.3390/v15081728/s1, Figure S1: Individual map of minor variant genomes across the SARS-CoV-2 genome for S1 (A), S2 (B), S3 (C), S4 (D), S5 (E), S6 (F), S7 (G), S8 (H), S9 (I), S10 (J), S11 (K), S12 (L), S13 (M), S14 (N), S15 (O), and S16 (P) in Figure 1; Figure S2: Non-synonymous substitutions in the minor genomic variants of SARS-CoV-2 from each of the 16 down selected patients focusing on sites that define VoCs (https://covariants.org/variants, accessed on 1 May 2022) in the spike protein (A) and other regions of the genome (B); Figure S3: Non-synonymous substitutions in the minor genomic variants of SARS-CoV-2 from each of the 16 down selected patients focusing on sites that define VoCs (https://covariants.org/variants, accessed on 1 May 2022) in the spike protein (A) and other regions of the genome (B); Figure S4: The difference in non-synonymous mutation frequencies between the inoculum and the days post-infection was examined in two non-human primate models: rhesus (R) and cynomolgus (C) macaques; Figure S5: The difference in synonymous mutation frequencies between the inoculum and the days post-infection was examined in two non-human primate models: rhesus (R) and cynomolgus (C) macaques.
