# Supplementary material for: Analysis of SARS-CoV-2 Population Genetics from Samples Associated with Huanan Market and Early Cases Identifies Substitutions Associated with Future Variants of Concern
Source: Viruses. 2023 Aug 12;15(8):1728. doi: 10.3390/v15081728 (PMC10459715; doi:10.3390/v15081728)
Supplement: Supplementary file 1 [file viruses-15-01728-s001.zip › viruses-2482663-supplementary.pdf]

Supplementary information

Fig. S1-S5

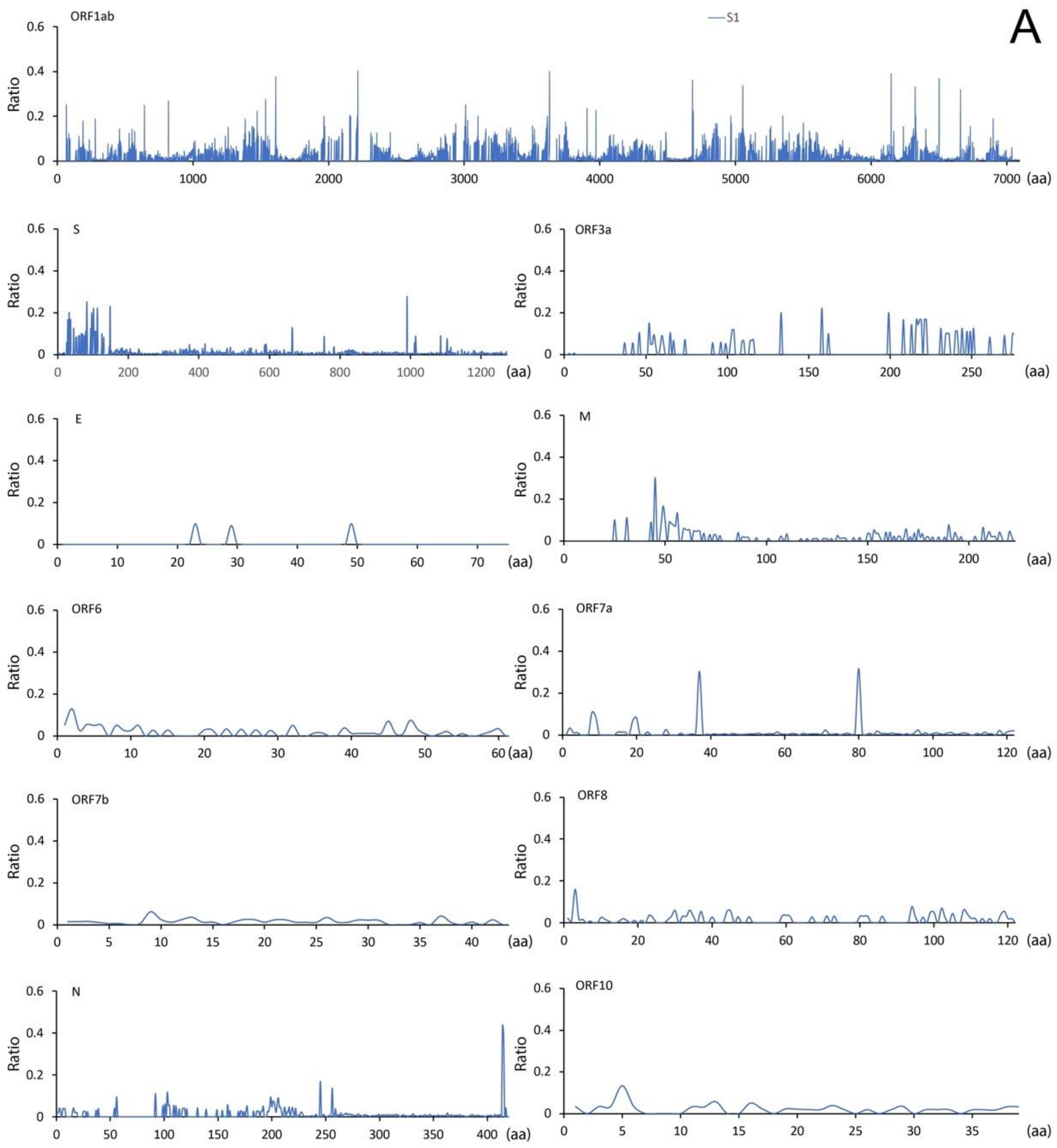
A
S1
ORF1ab
S
ORF3a
E
M
ORF6
ORF7a
ORF7b
ORF8
N
ORF10
Ratio
(aa)

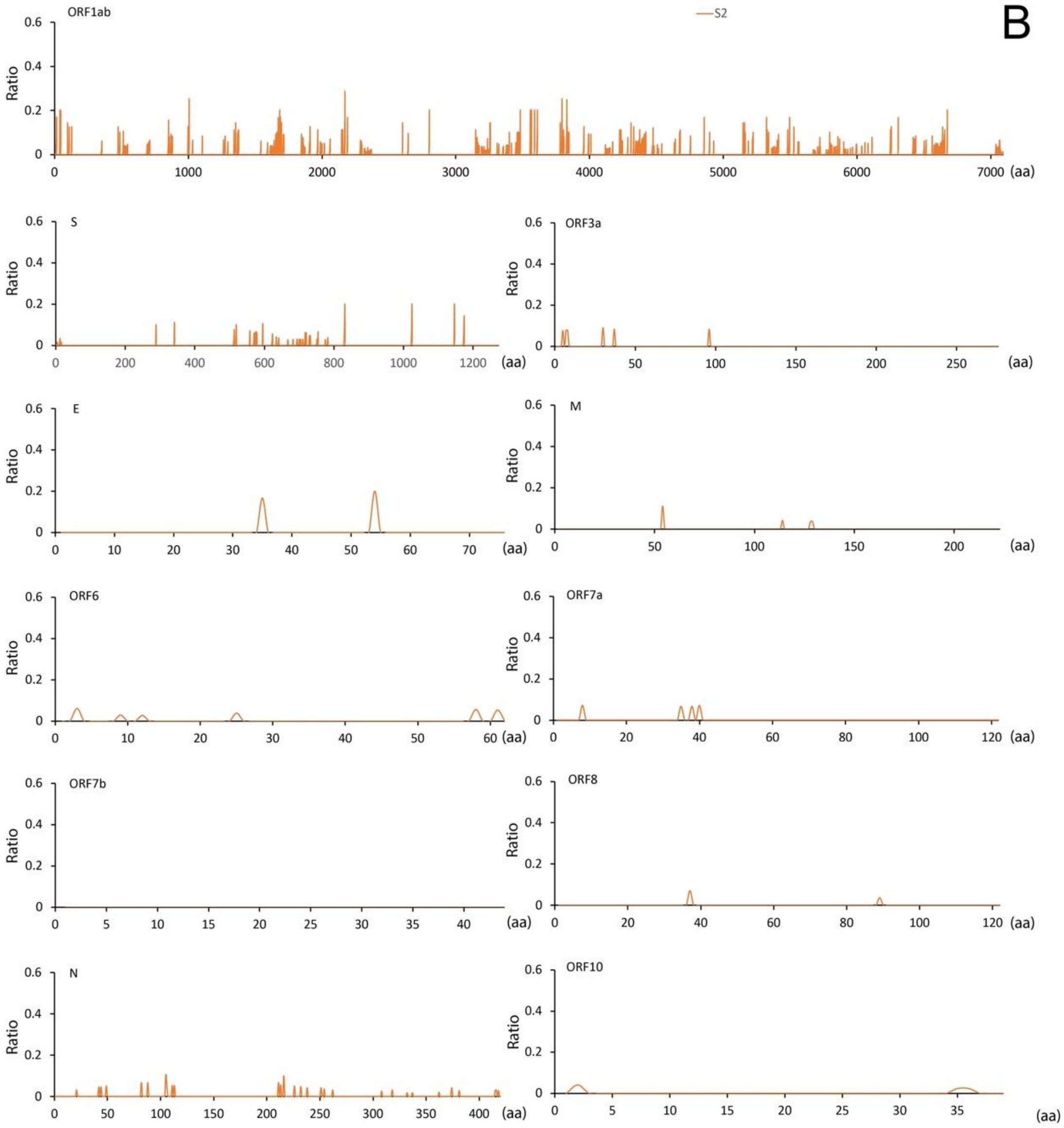
B
S2
ORF1ab
S
ORF3a
E
M
ORF6
ORF7a
ORF7b
ORF8
N
ORF10
Ratio
(aa)

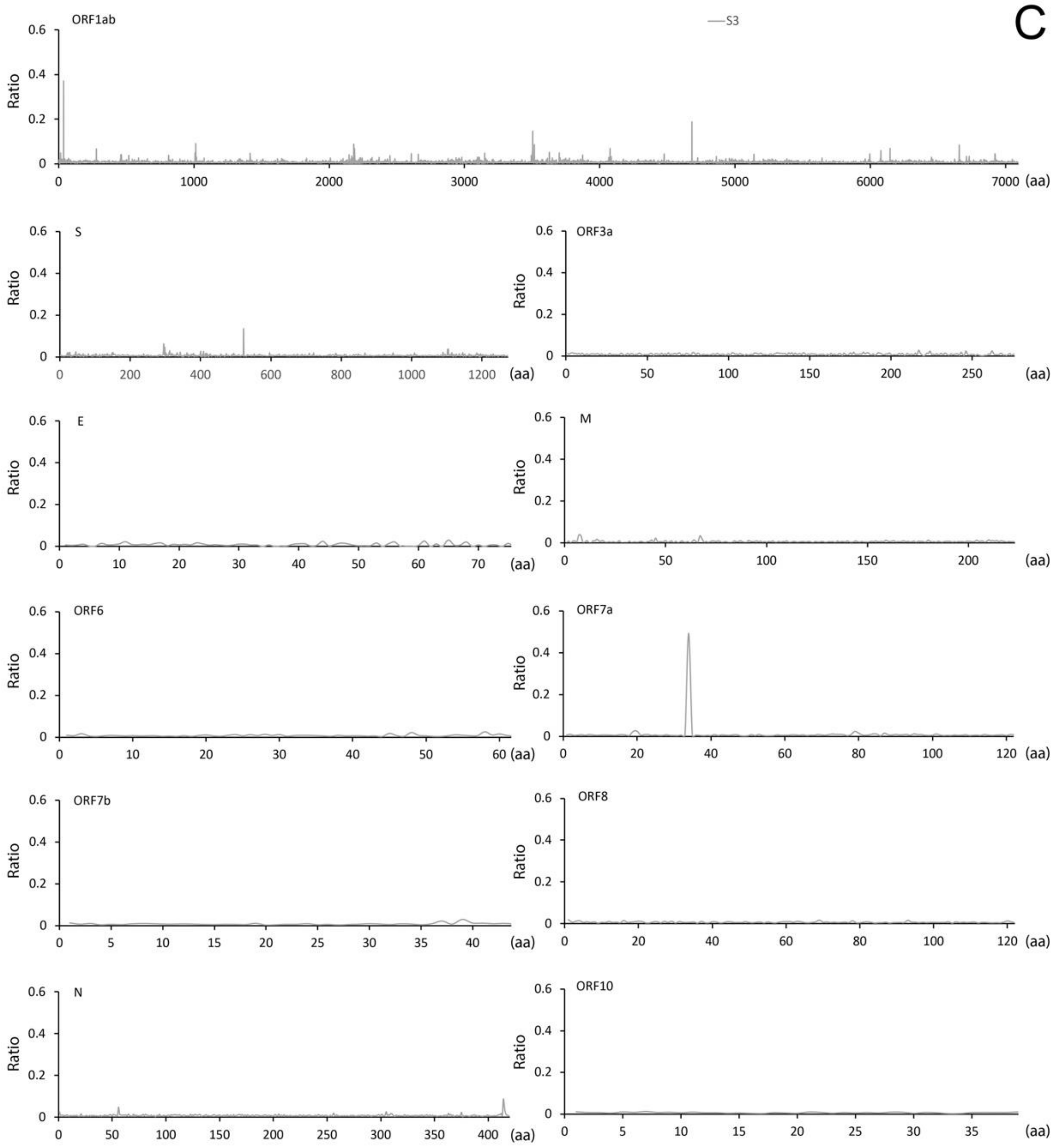

C
S3
ORF1ab
S
ORF3a
E
M
ORF6
ORF7a
ORF7b
ORF8
N
ORF10
Ratio
(aa)

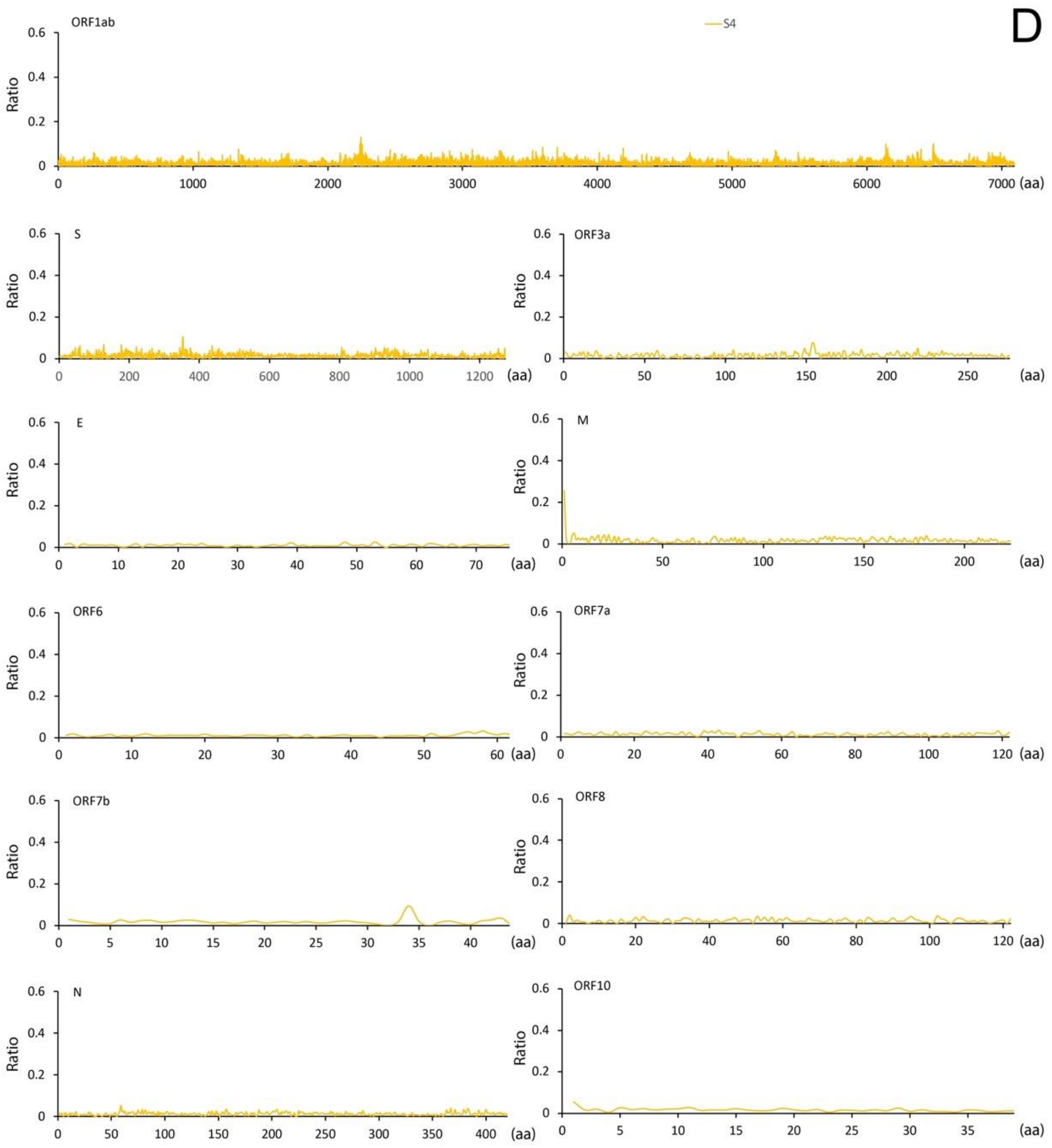
D
S4
ORF1ab
S
ORF3a
E
M
ORF6
ORF7a
ORF7b
ORF8
N
ORF10
Ratio
(aa)

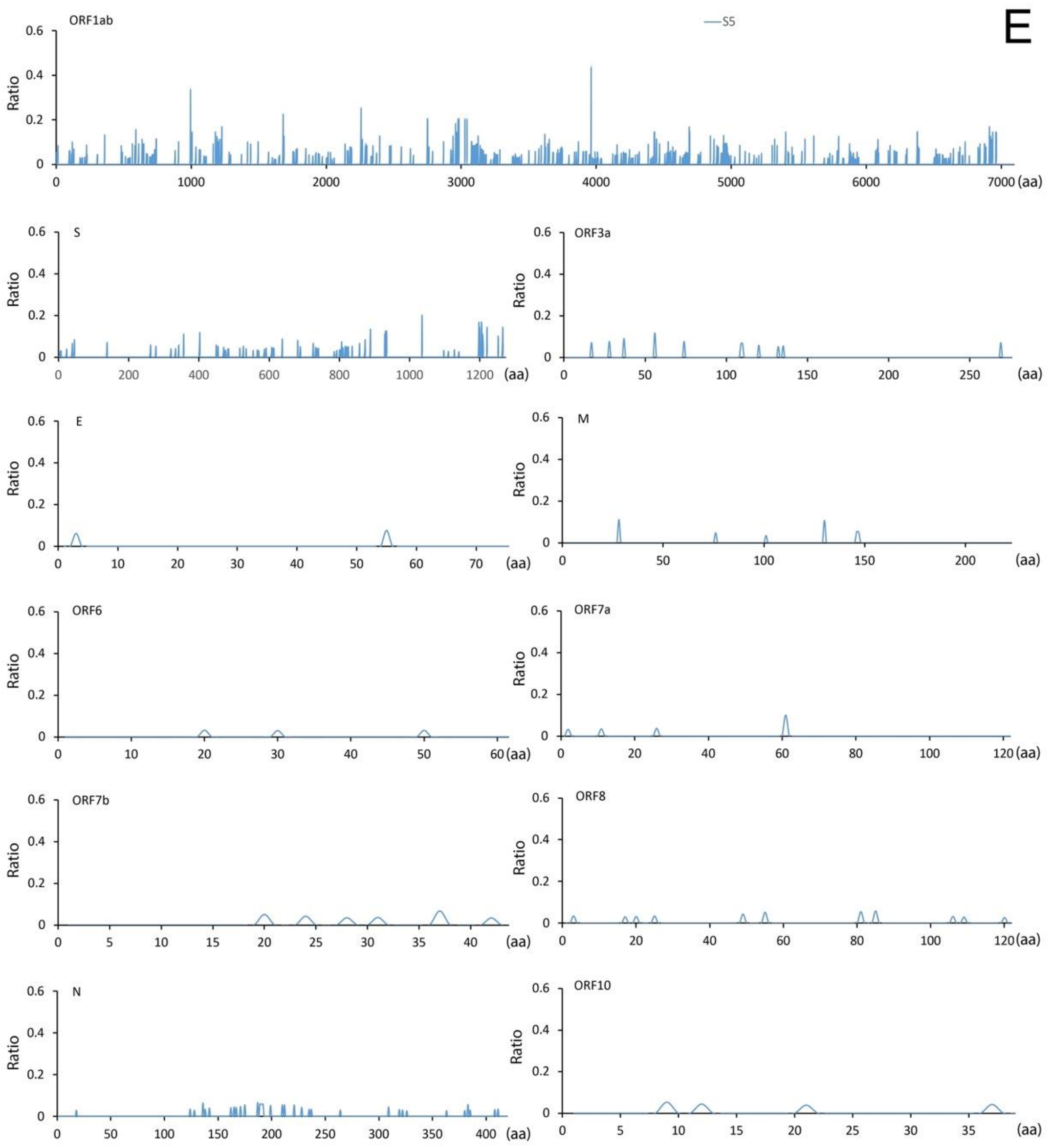

E
S5
ORF1ab
S
ORF3a
E
M
ORF6
ORF7a
ORF7b
ORF8
N
ORF10
Ratio
(aa)

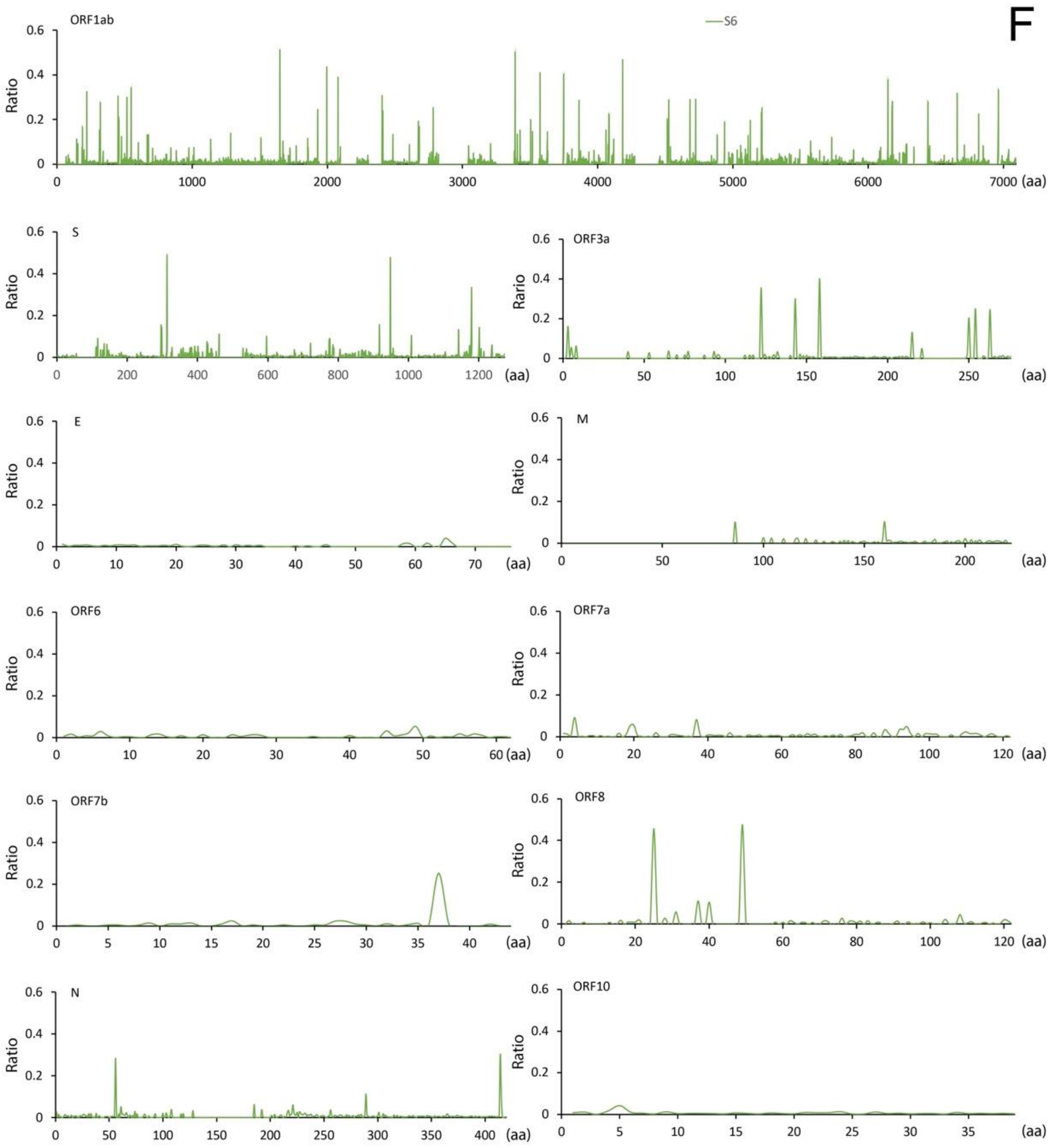

F
S6
ORF1ab
S
ORF3a
E
M
ORF6
ORF7a
ORF7b
ORF8
N
ORF10
Ratio
(aa)

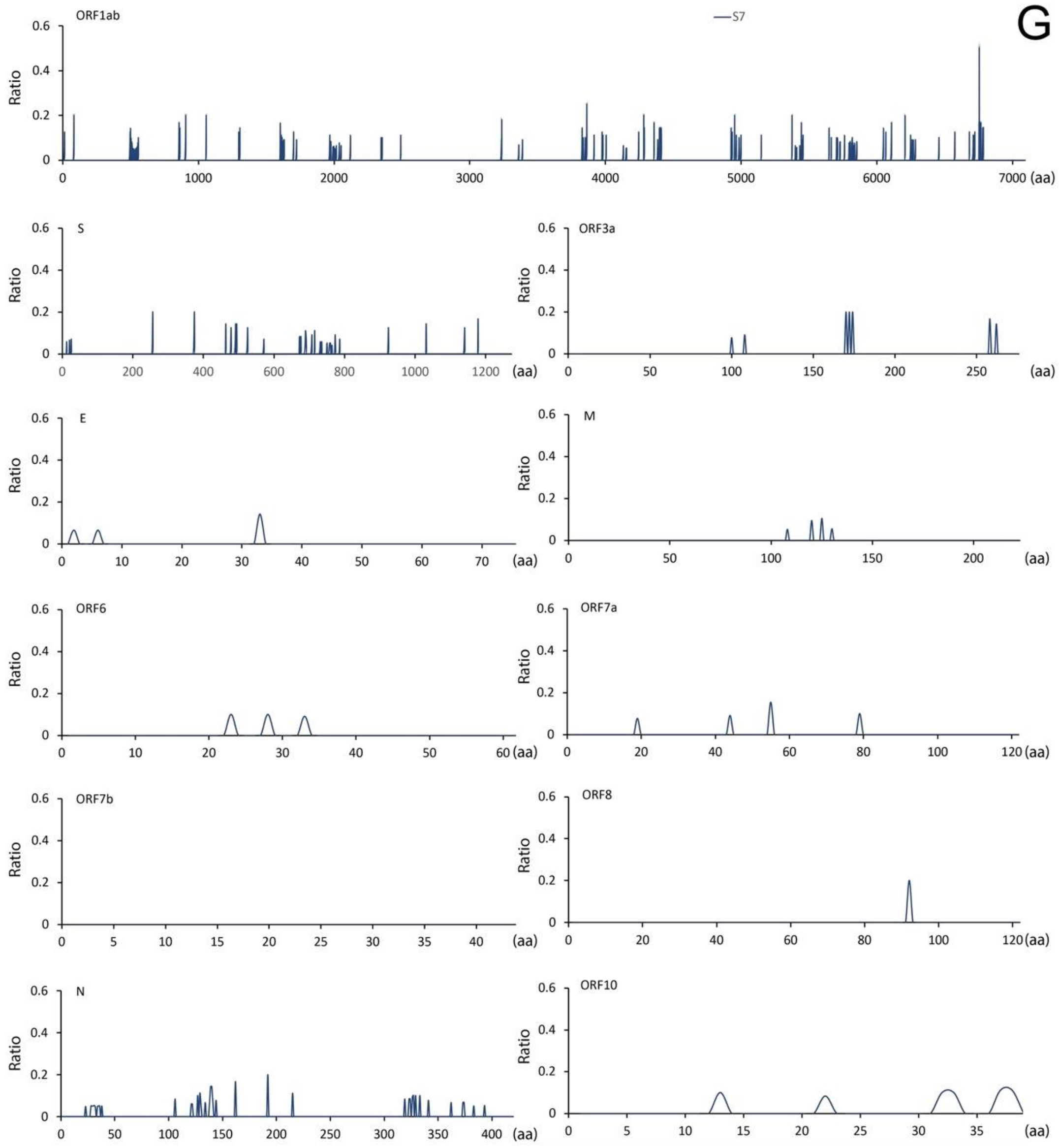
G
S7
ORF1ab
S
ORF3a
E
M
ORF6
ORF7a
ORF7b
ORF8
N
ORF10
Ratio
(aa)

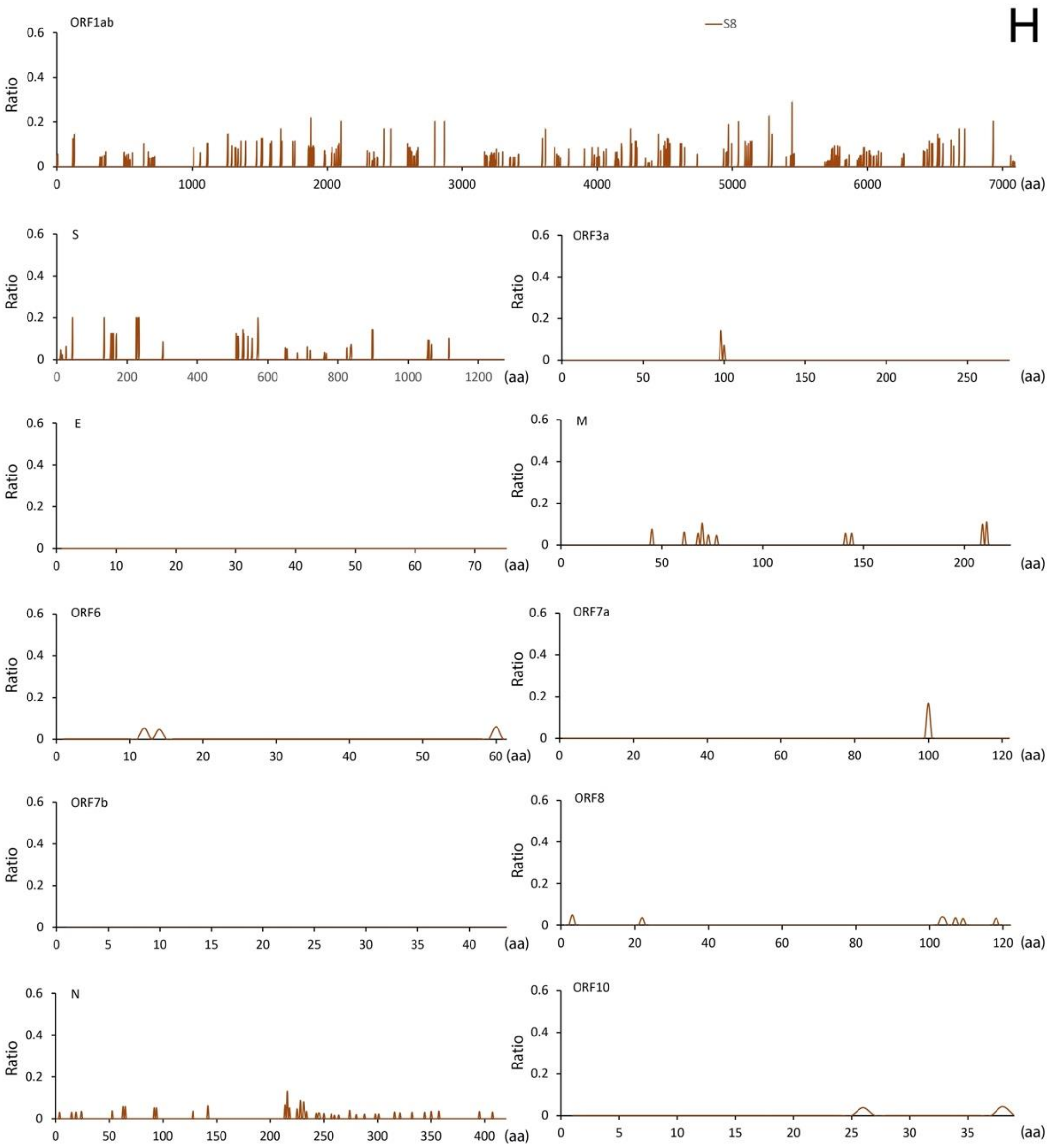

H
S8
ORF1ab
S
ORF3a
E
M
ORF6
ORF7a
ORF7b
ORF8
N
ORF10
Ratio
(aa)

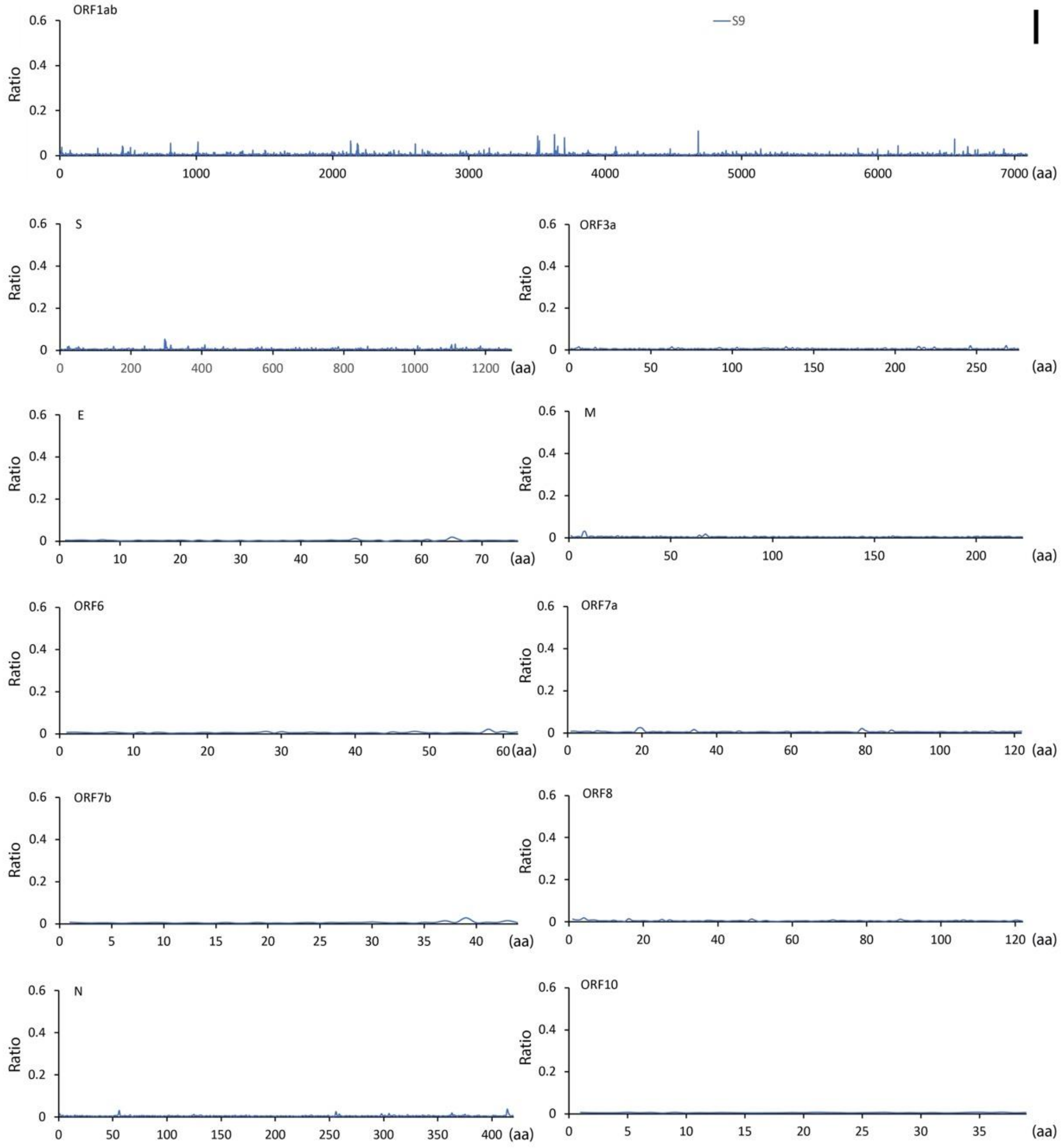
ORF1ab
S9
I
S
ORF3a
E
M
ORF6
ORF7a
ORF7b
ORF8
N
ORF10
Ratio
(aa)

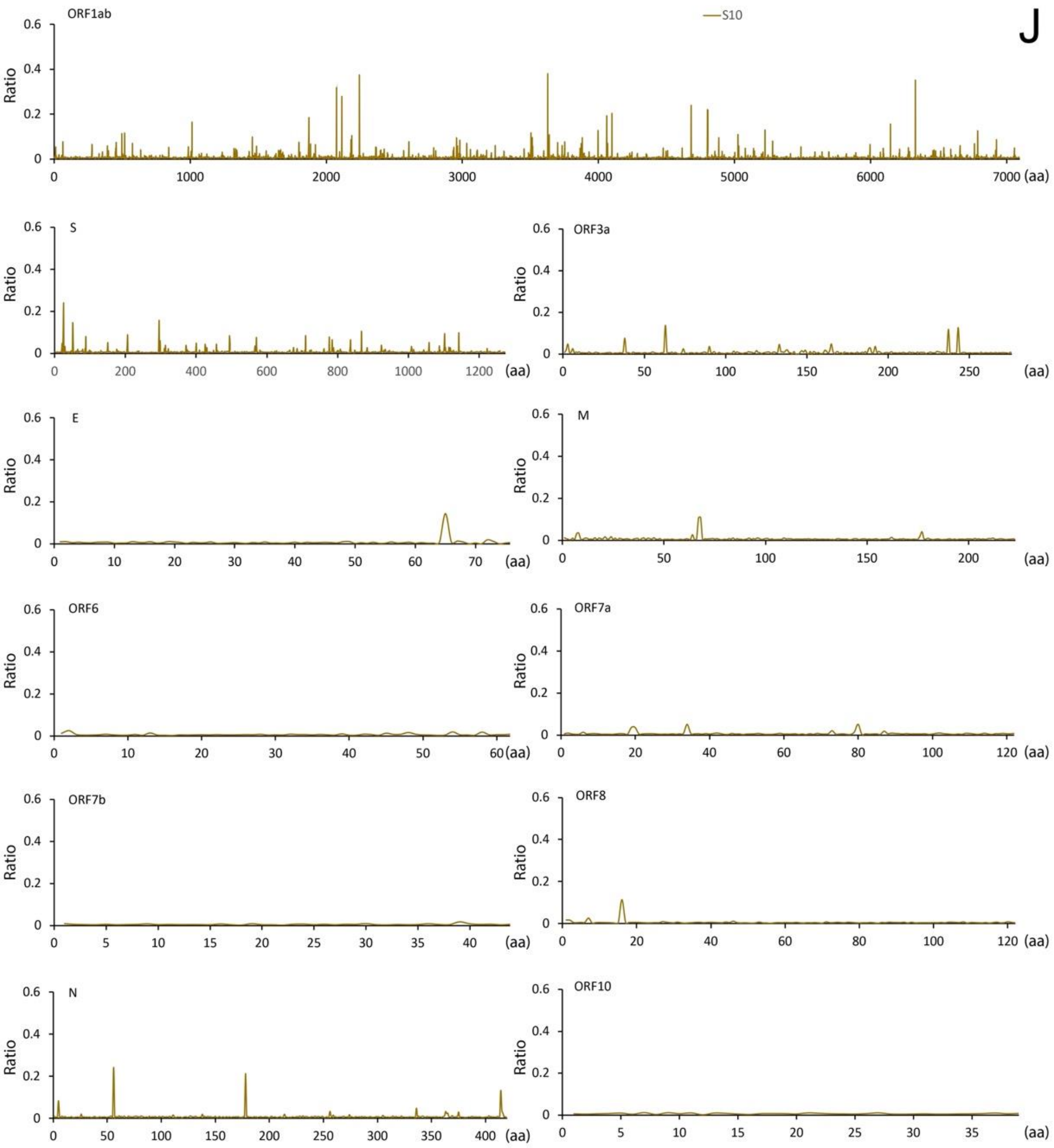
J
S10
ORF1ab
S
ORF3a
E
M
ORF6
ORF7a
ORF7b
ORF8
N
ORF10
Ratio
(aa)

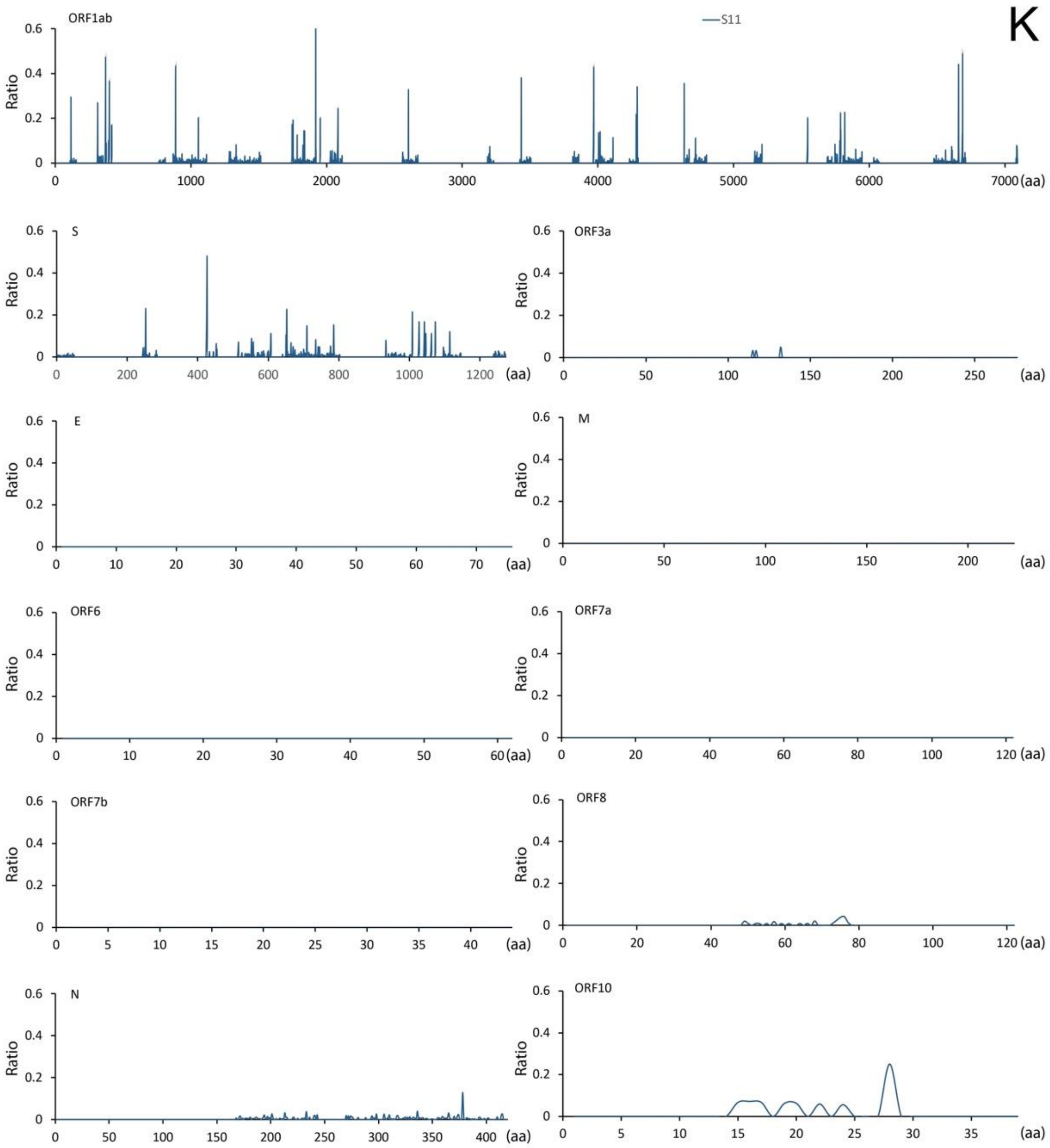

K
S11
ORF1ab
S
ORF3a
E
M
ORF6
ORF7a
ORF7b
ORF8
N
ORF10
Ratio
(aa)

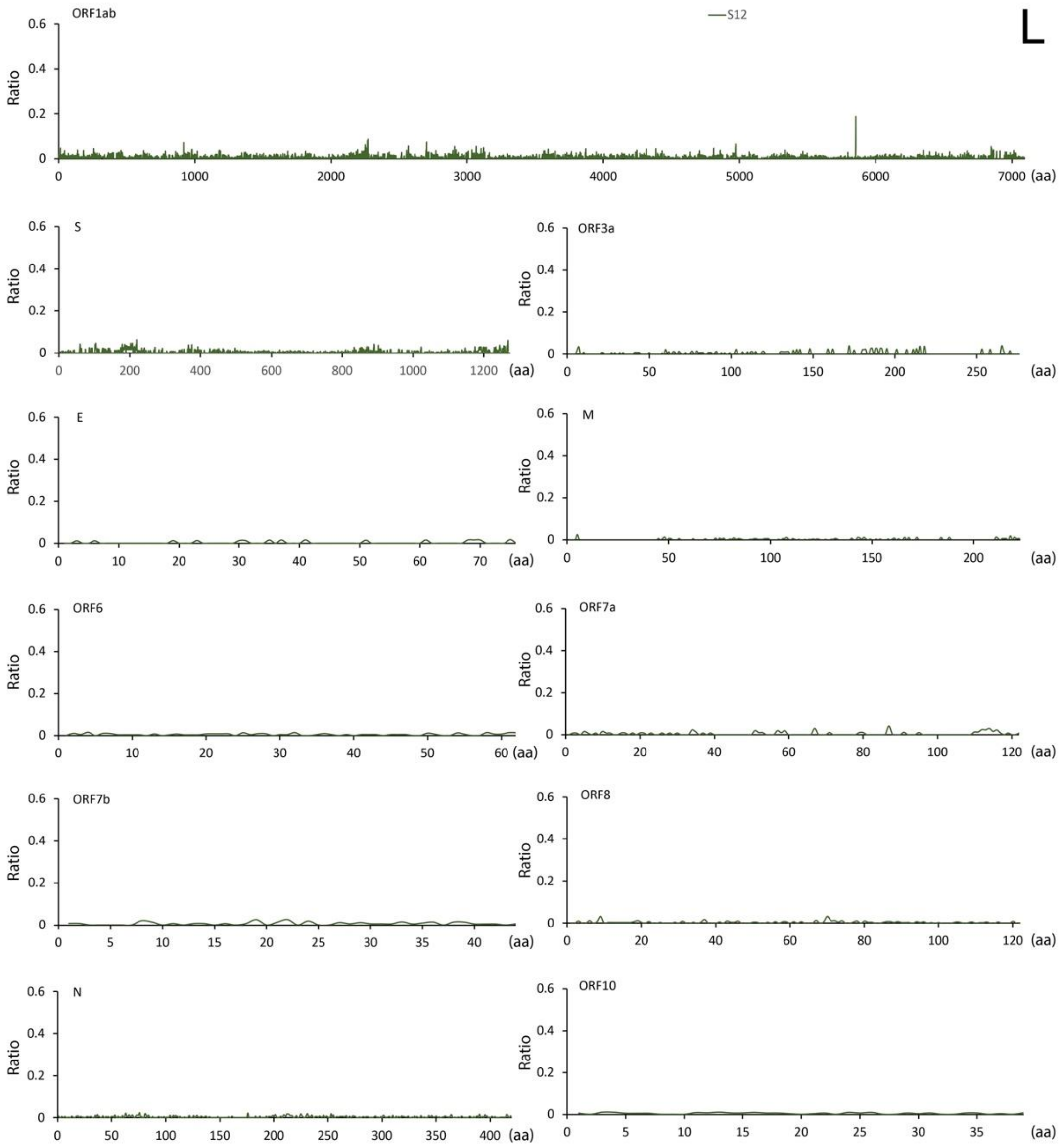
L
S12
ORF1ab
S
ORF3a
E
M
ORF6
ORF7a
ORF7b
ORF8
N
ORF10
Ratio
(aa)

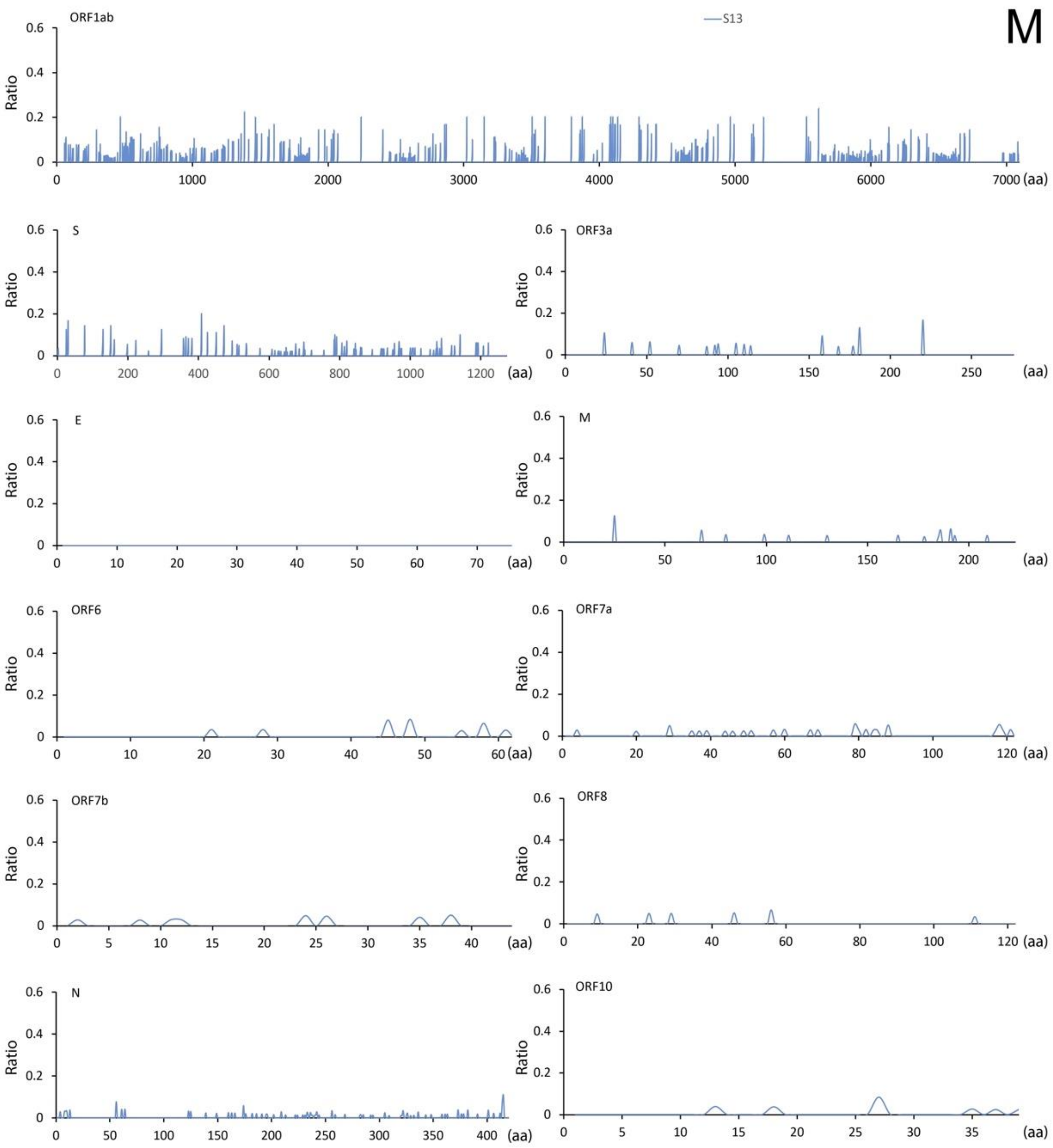
M
S13
ORF1ab
S
ORF3a
E
M
ORF6
ORF7a
ORF7b
ORF8
N
ORF10
Ratio
(aa)

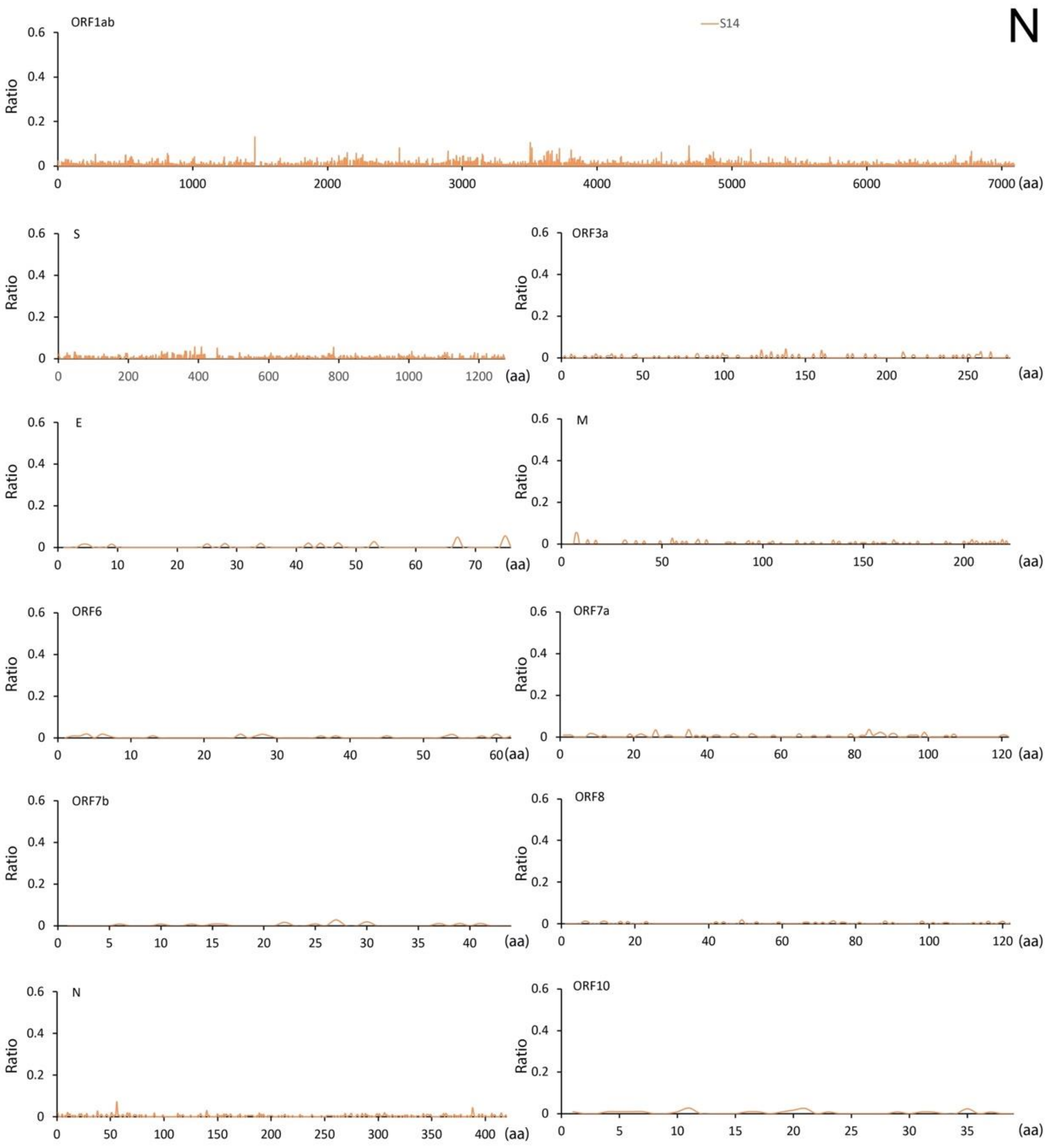
N
S14
ORF1ab
S
ORF3a
E
M
ORF6
ORF7a
ORF7b
ORF8
N
ORF10
Ratio
(aa)

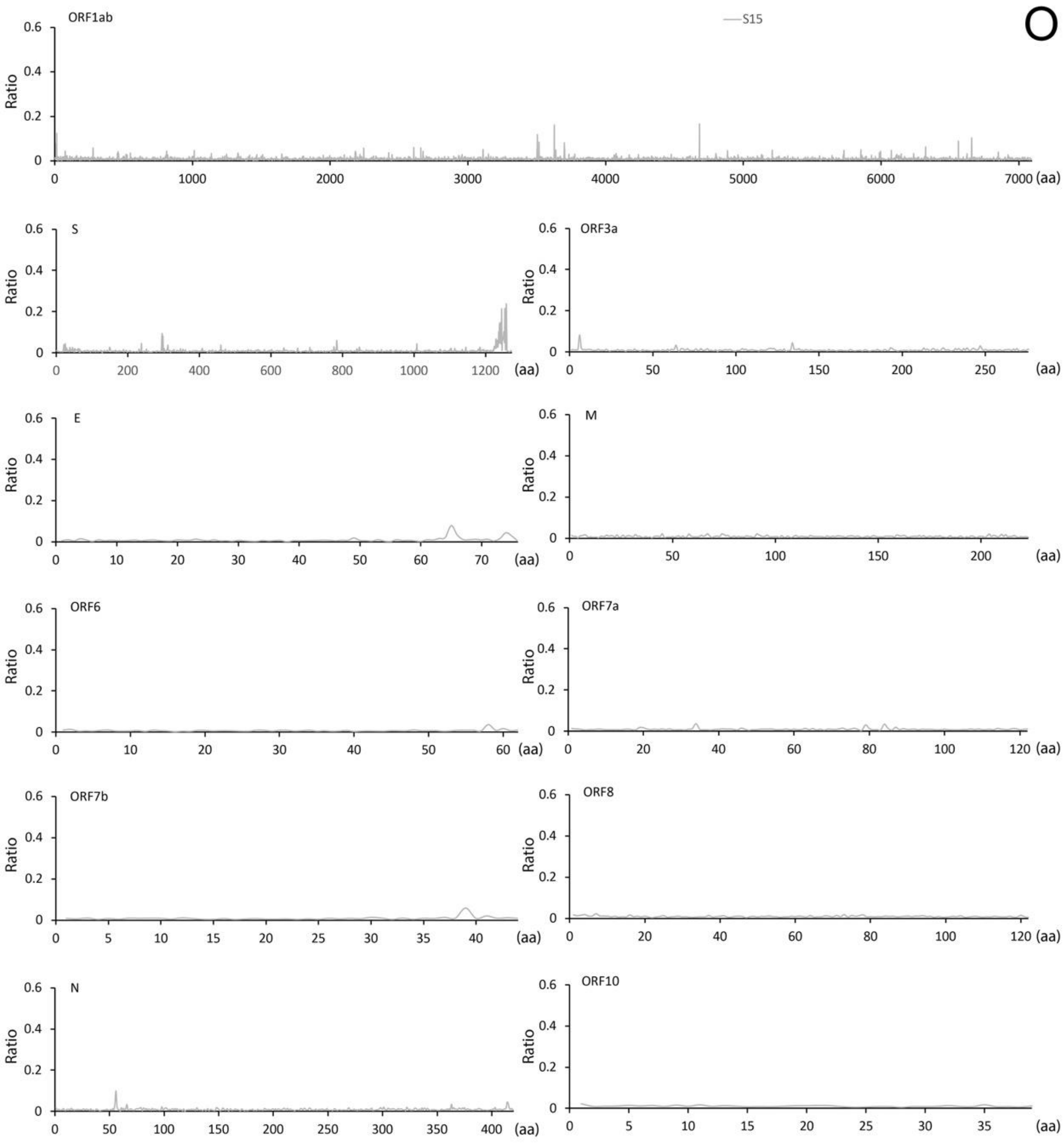

O
S15
ORF1ab
S
ORF3a
E
M
ORF6
ORF7a
ORF7b
ORF8
N
ORF10
Ratio
(aa)

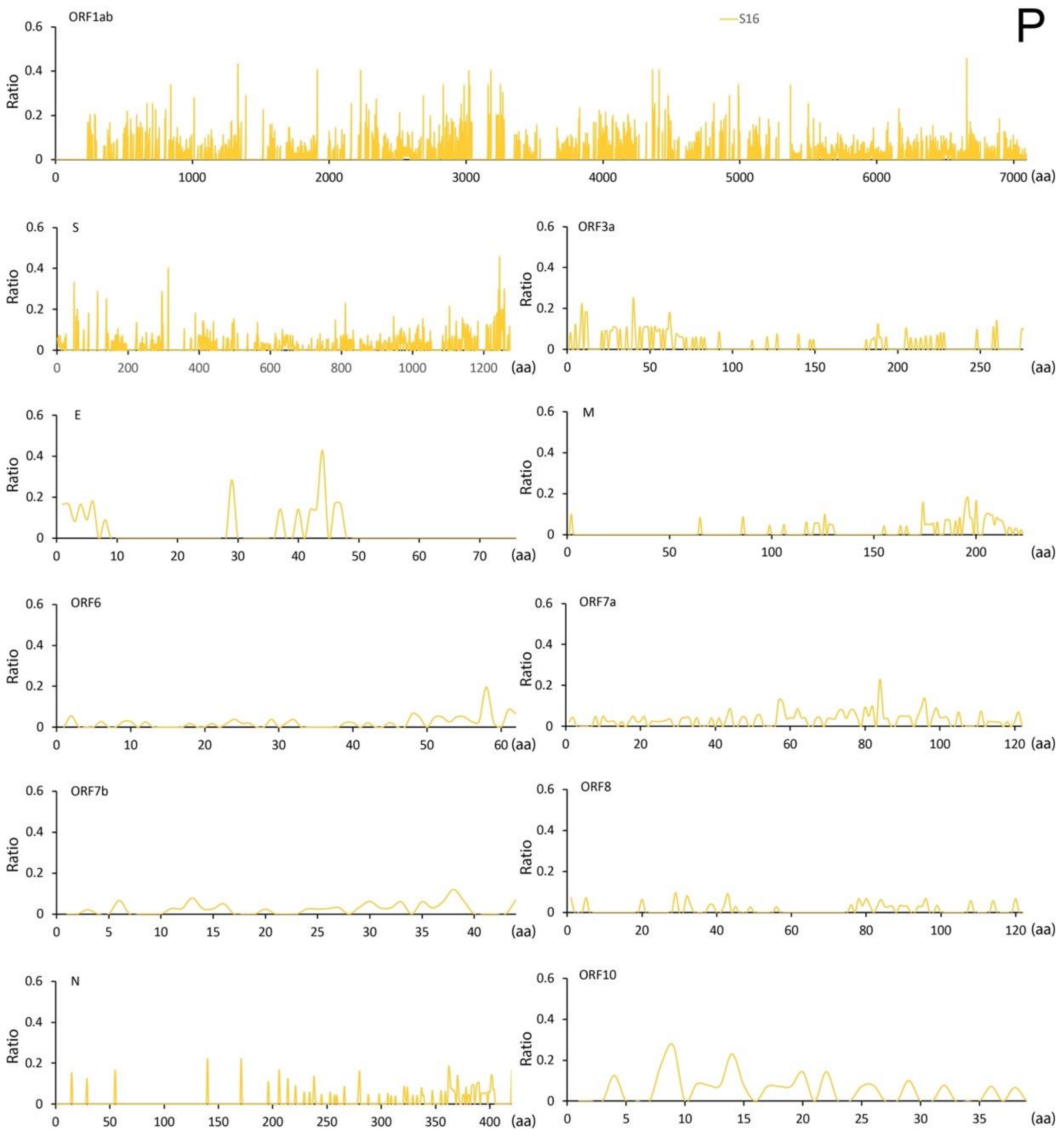

Fig. S1. Individual map of minor variant genomes across the SARS-CoV-2 genome for S1 (A), S2 (B), S3 (C), S4 (D), S5 (E), S6 (F), S7 (G), S8 (H), S9 (I), S10 (J), S11 (K), S12 (L), S13 (M), S14 (N), S15 (O), and S16 (P) in Fig. 1. The amino acid site with coverage >= 10 were showed.

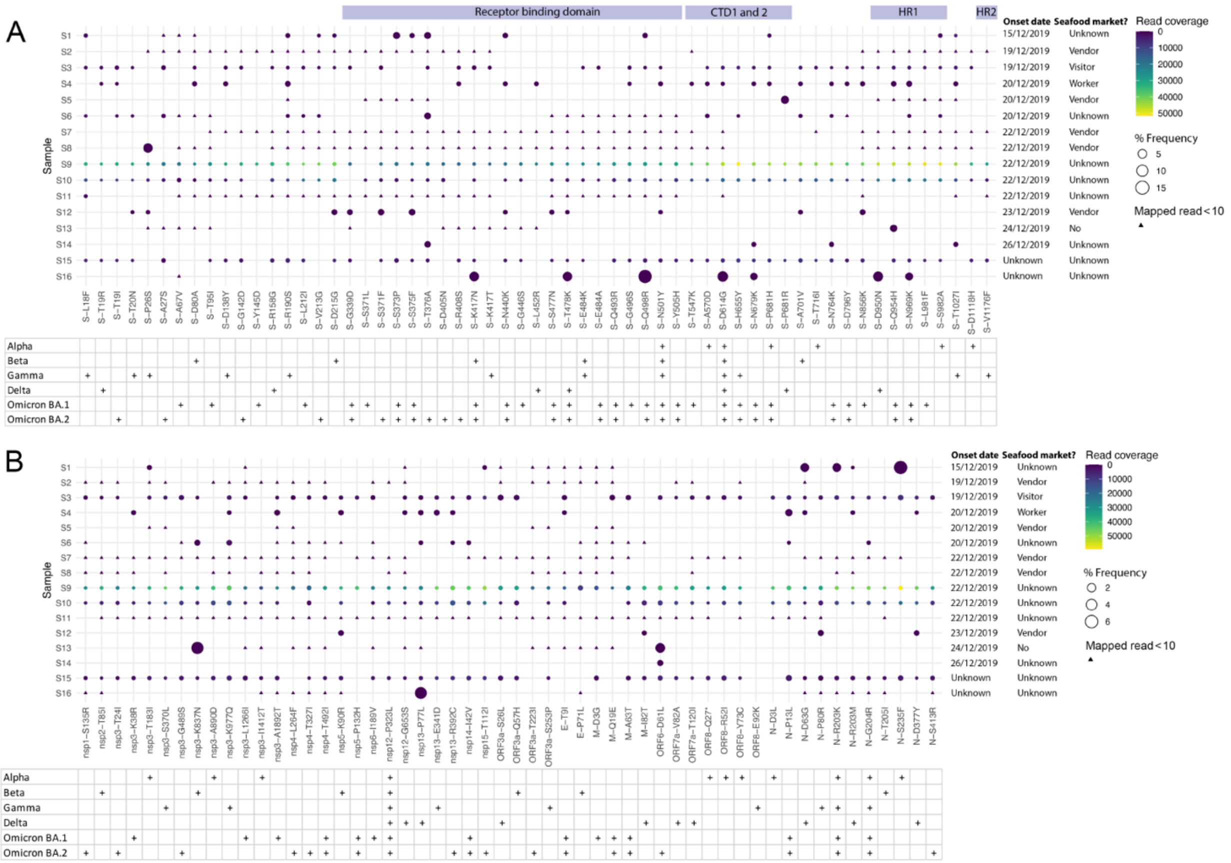

Fig. S2. Non-synonymous substitutions in the minor genomic variants of SARS-CoV-2 from each of the 16 down selected patients focusing on sites that define VoCs (https://covariants.org/variants) in the spike protein (A) and other regions of the genome (B). The amino acid site with coverage >= 10 were showed.

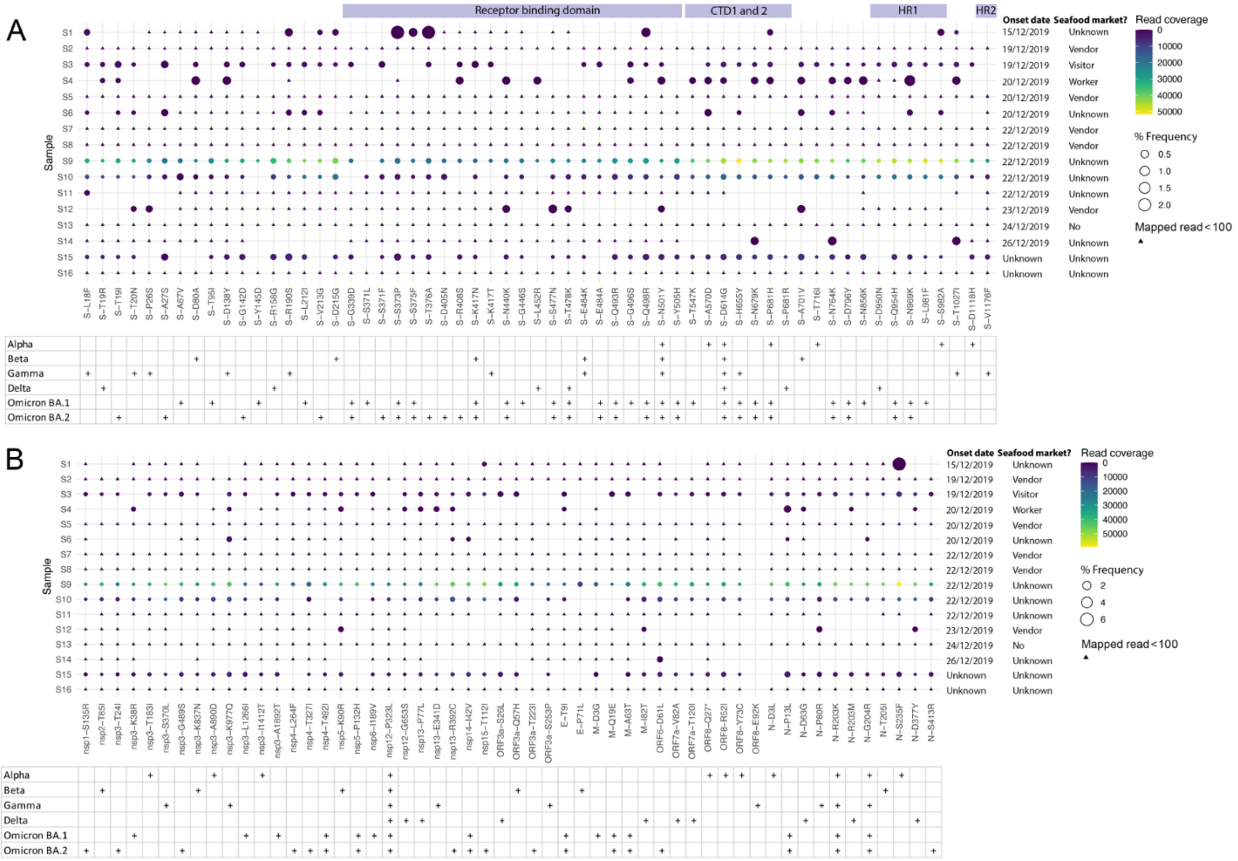

Fig. S3. Non-synonymous substitutions in the minor genomic variants of SARS-CoV-2 from each of the 16 down selected patients focusing on sites that define VoCs (https://covariants.org/variants) in the spike protein (A) and other regions of the genome (B). The amino acid site with coverage >= 100 were showed.

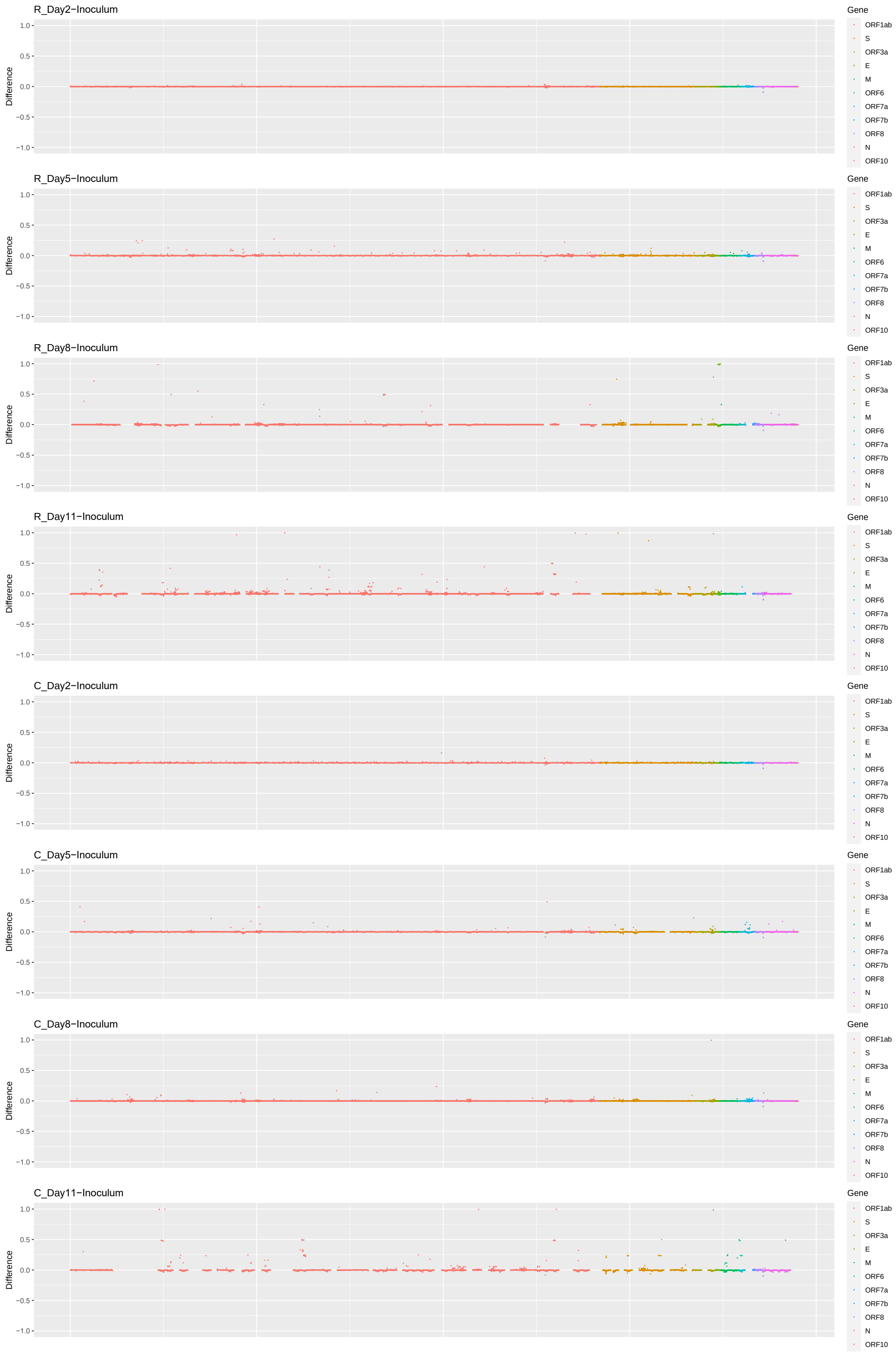

Fig. S4. The difference in non-synonymous mutation frequencies between the inoculum and the days post-infection was examined in two non-human primate models: rhesus (R) and cynomolgus (C) macaques. Each dot represents an amino acid site. Positive values indicate an increase in mutation frequencies compared to the inoculum during the days post-infection. The data were extracted from a published paper (DOI: 10.1186/s13059-023-02881-5).

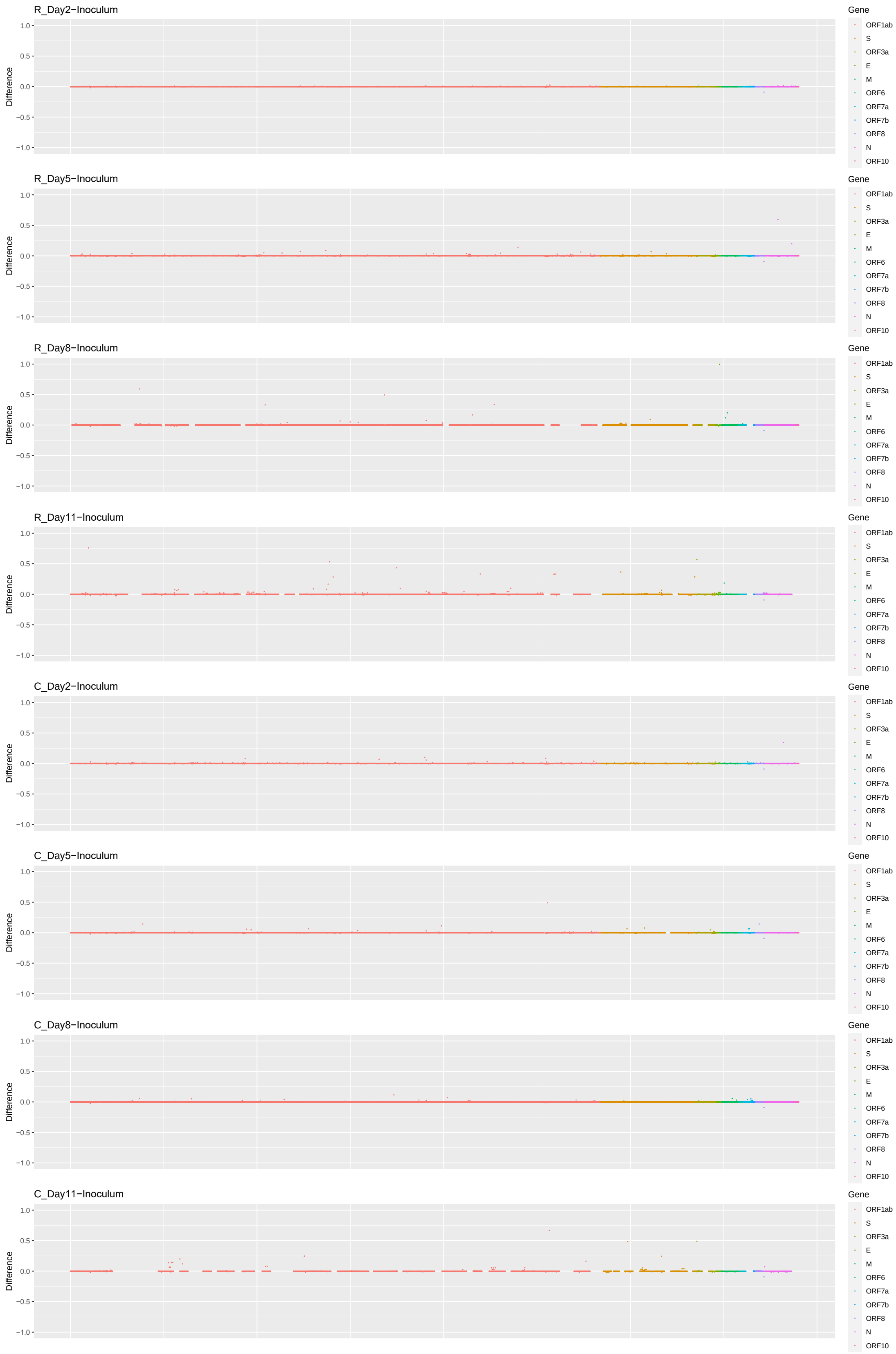

Fig. S5. The difference in synonymous mutation frequencies between the inoculum and the days post-infection was examined in two non-human primate models: rhesus (R) and cynomolgus (C) macaques. Each dot represents an amino acid site. Positive values indicate an increase in mutation frequencies compared to the inoculum during the days post-infection. The data were extracted from a published paper (DOI: 10.1186/s13059-023-02881-5).
